# Hyperactivity of the non-canonical inflammasome in SPG11 and SPG48

**DOI:** 10.1016/j.ebiom.2025.105985

**Published:** 2025-10-24

**Authors:** Muhammad Awais Afzal, Mohamed Ghait, Adeela Hussain, Anke Siegmund, Lorena Tuchscherr, Petra Babic, Adrian T. Press, Robert Hardt, Dominic Winter, Annekathrin Rödiger, Rebecca Schüle, Jens Fielitz, Michael Bauer, Christian Andreas Hübner

**Affiliations:** a Institute of Human Genetics, Jena University Hospital, Friedrich Schiller University, Am Klinikum 1, 07747, Jena, Germany; b DZHK (German Center for Cardiovascular Research), Partner Site Greifswald, 17475, Greifswald, Germany; c Institute of Medical Microbiology, Jena University Hospital, 07747, Jena, Germany; d Department of Anesthesiology and Intensive Medicine, Jena University Hospital, 07747, Jena, Germany; e Seer Bio GmbH - STAC Europe, Venusberg-Campus 1, 53127, Bonn, Germany; f Department Metabolism, Senescence and Autophagy, Research Center One Health Ruhr, University Alliance Ruhr, University Hospital Essen, Medical Faculty, University of Duisburg-Essen, 45147, Essen, Germany; g Department of Neurology, Neuromuscular Center, Jena University Hospital, Germany; h Division of Neurodegenerative Diseases and Movement Disorders, Department of Neurology, Heidelberg University Hospital and Faculty of Medicine, Heidelberg, Germany; i Center for Sepsis Control and Care (CSCC), Jena University Hospital, 07747, Jena, Germany; j Department of Internal Medicine B, Cardiology, University Medicine Greifswald, 17475, Greifswald, Germany; k Friedrich Schiller University, Medical Faculty, Kastanienstr. 1, 07747, Jena, Germany; l Institute of Biochemistry and Molecular Biology, University of Bonn, 53115, Bonn, Germany; m Center for Rare Diseases, Jena University Hospital, Friedrich Schiller University, Am Klinikum 1, 07747, Jena, Germany; n Department of Neurodegenerative Diseases, Hertie Center for Neurology, University of Tübingen, Tübingen, Germany

**Keywords:** HSP, Inflammasome, AP-5, SPG11, SPG48, Neurodegeneration

## Abstract

**Background:**

Hereditary spastic paraplegia (HSP) denotes a heterogeneous group of neurodegenerative spastic gait disorders. Variants in *SPG11* cause the most common autosomal recessive HSP also known as SPG11. The gene product Spatacsin interacts with the adaptor protein complex 5 (AP5). Because neurodegeneration in SPG11 is accompanied by marked neuro-inflammation, we hypothesised that Spatacsin may play a cell-autonomous role in pro-inflammatory cells.

**Methods:**

Inflammasome activation was assessed in primary microglia and bone-marrow-derived-macrophages (BMDMs) from wild-type, *Spg11,* and *Ap5z1* knockout (KO) mice and monocyte-derived-macrophages (MDMs) from patients with *SPG11* mutations. Wild-type and *Spg11* KO mice were used to study microglia activation and LPS-induced inflammatory responses *in vivo*.

**Findings:**

We show that microglia activation is more pronounced in pre-symptomatic *Spg11* KO compared with control mice following systemic lipopolysaccharide (LPS) challenge. Our subsequent studies demonstrate that the activation of the non-canonical inflammasome results in a stronger inflammatory response in primary microglia and BMDMs from *Spg11* KO mice, while the canonical pathway is unaffected. These findings are also observed in MDMs isolated from patients carrying loss-of-function *SPG11* mutations. *In vivo*, LPS triggers a much stronger inflammatory response and leads to drastically increased lethality in *Spg11* KO mice. Mass spectrometry of activated BMDMs unveils a massive downregulation of AP5 subunits upon disruption of *Spg11*. Notably, the disruption of its ζ-subunit Ap5z1, which is associated with SPG48, also sensitises the non-canonical inflammasome.

**Interpretation:**

Our findings suggest that a hyper-reactivity of the non-canonical inflammasome in innate immune cells contributes to neuro-inflammation in SPG11 and SPG48.

**Funding:**

Please see Acknowledgements.

**Research in context**

**Evidence before this study**

Post-mortem brains from patients with SPG11 exhibited pronounced neuron loss with microgliosis. Patient-derived cells differentiate into microglia displayed increased proinflammatory cytokine release and neurotoxicity.

**Added value of this study**

Here, we show that disruption of *SPG11* results in a gain-of-function of the non-canonical inflammasome in microglia and macrophages while leaving the canonical pathway unaffected. Consequently, the inflammatory response to LPS is increased in *Spg11* KO mice. The downregulation of AP5 subunits in *Spg11* KO mice and the sensitisation of the non-canonical inflammasome in *Ap5z1* KO mice link AP5 with the regulation of the non-canonical inflammasome.

**Implications of all the available evidence**

Our study shows a sensitisation of the non-canonical inflammasome in SPG11 and SPG48 with consequences for the response to proinflammatory triggers, which promotes neuroinflammation in SPG11. Therefore, studies are warranted to assess whether anti-inflammatory drugs may mitigate disease progression in patients with either SPG11 or SPG48. Moreover, our findings suggest that patients with either SPG11 or SPG48 may be prone to infection-triggered complications.

## Introduction

Hereditary spastic paraplegia (HSP) is defined by the length-dependent degeneration of corticospinal tract axons thus causing progressive spasticity and weakness of predominantly lower extremities.^1^^,^^2^ To date more than 80 different genetic entities can be distinguished, which are denoted as SPGs with a running number. Additional symptoms such as cognitive impairment, cerebellar ataxia, epilepsy, retinal degeneration, lower motor neuron damage, and thinning of the corpus callosum characterise spastic paraplegia type-11 (*SPG11* or also known as *KIAA1840*) and spastic paraplegia type-15 (*SPG15*). These common autosomal recessive SPGs are caused by mutations in *SPG11* and *ZFYVE26*, respectively.^3^, ^4^, ^5^, ^6^ The corresponding gene products, Spatacsin and Spastizin, are broadly expressed and interact with the adaptor protein complex 5 (AP5), a heterotetramer with only partially known functions in vesicle trafficking.^7^^,^^8^ Notably, mutations of its ζ subunit are associated with SPG48, which shares several features with SPG11 and SPG15.^9^

Both Spatacsin and Spastizin are important for the recycling of lysosomes from autolysosomes, a process also known as autophagic lysosome reformation.^10^^,^^11^ Disease causing mutations thus compromise autophagy and result in the accumulation of intracellular auto-fluorescent debris, which in the long-term leads to neuron loss.^5^^,^^11^^,^^12^ Remarkably, genetic ablation of lymphocytes or immuno-modulators attenuated neurological deterioration in a mouse model for SPG11.^13^^,^^14^ To date, only a few patients with SPG11 have been examined neuropathologically. Apart from a strong infiltration of brain tissue by immune cells, with a marked increase of activated IBA1-positive microglia,^13^ infiltration of myelin-loaded macrophages due to myelin damage has also been reported.^15^

Microglia are the resident macrophages of the central nervous system (CNS) and play a crucial role for neuroinflammation. Specific cytosolic pattern recognition receptors (PRRs), which sense microbe-derived pathogen-associated molecular patterns (PAMPs), and damage-associated molecular patterns (DAMPs) from the host cell can lead to the activation of microglia by triggering either canonical or non-canonical multi-protein signalling complexes also known as NLRP3 (NOD-, LRR- and pyrin domain-containing protein 3) inflammasomes.^16^^,^^17^ Activated inflammasomes then proteolytically cleave pro-inflammatory cytokines, interleukin 1β (IL-1β) and interleukin 18 (IL-18), as well as the pore-forming molecule Gasdermin D (GSDMD). This induces a pro-inflammatory form of programmed cell death, referred to as pyroptosis, characterised by loss of membrane integrity.^18^ Pyroptosis of immune and inflammatory cells can limit intracellular pathogen replication and activate other immune cells to phagocytose and eliminate pathogens.^19^^,^^20^ However, excessive pyroptosis can also perpetuate inflammation by triggering a massive release of proinflammatory cytokines^17^^,^^21^ and lead to immunosuppression.^22^^,^^23^ Pyroptosis-induced organ dysfunction is one of the main clinical challenges during endotoxemia and sepsis. Severely affected organs include the nervous, cardiovascular, and respiratory system as well as kidneys and liver.^24^, ^25^, ^26^, ^27^, ^28^

Given the growing recognition that immune dysregulation contributes to the pathophysiology of SPG11,^13^^,^^14^^,^^29^ we hypothesised that disruption of Spatacsin may alter inflammatory responses of innate immunocompetent cells. Here, we challenged young asymptomatic *Spg11* KO mice^11^ without overt microglia activation with Lipopolysaccharide (LPS) to trigger systemic inflammation. We observed that disruption of *Spg11* affects microglia activation in a cell-autonomous manner. While cytokine release from primary *Spg11* KO microglia and bone-marrow-derived-macrophages (BMDMs) did not differ upon activation of the canonical inflammasome pathway, it was strongly increased upon activation of the non-canonical pathway. This also applied to BMDMs deficient for Ap5z1 suggesting that AP5 is involved in the deregulation of the non-canonical inflammasome pathway. When we challenged *Spg11* KO mice with *E. coli* LPS, inflammation and lethality was drastically increased compared with controls. Our findings are also relevant for patients as cytokine release from monocyte-derived-macrophages (MDMs) from patients with SPG11 was strongly increased upon activation of the non-canonical inflammasome pathway.

This study provides an explanation as to why anti-inflammatory agents should be considered in the treatment of patients suffering from SPG11 or SPG48. Moreover, the dysregulation of the non-canonical inflammasome pathway poses patients with SPG11 or SPG48 at risk for severe systemic inflammation. Our data are likely also valid for patients with SPG15, because Spastizin also interacts with Spatacsin and the AP5 complex.

## Methods

### Mouse experiments

All animal experiments were approved by the “*Thüringer Landesamt für Verbraucherschutz”*(TLV) and performed in accordance with the animal welfare regulations (licence numbers 02-027/16, UKJ-17-006 and UKJ-22-019). Mice were housed with a 12 h light/dark cycle and fed on a regular diet *ad libitum*. Experiments were performed on a mixed 129SvJ/C57BL/6 background and littermates were randomly assigned into experimental cohorts with comparable distribution by sex. Experiments were performed in a blinded manner at different ages as indicated. *Spg11* KO and *Ap5-z1* KO mice used in this study have been described previously.^8^^,^^11^ Studies involving humans were approved by the local Ethics committee.

### Microglia isolation and culture

Brains from WT and *Spg11* KO pups (P2–P4) were collected in a 6-well-plate containing pre-cold 1× PBS. Cerebral cortices were isolated, minced and digestion buffer (8 U/ml papain (Cell Systems, LK003176) + 125 U/ml DNase (ThermoFisher Scientific, 89836) in 1× DMEM (Gibco, 32430027)) added (0.5 ml/brain). The plate was incubated in a humidified chamber (5% CO2, 37 °C) for 20 min. The digestion was terminated by adding 3 ml of 1× DMEM (Gibco, 32430027) containing 10% FCS. Cells were gently pipetted up and down and the mixture transferred to a 15 ml falcon tube and settled for 2–3 min. The cell culture suspension was passed through a 70 μm cell strainer and collected in a 50 ml tube. 4–5 ml of fresh cell culture medium (1× DMEM (Gibco, 32430027) + 10% FBS (Biowest, S1810-500) + 100 U/ml Pen/Strep (Gibco, 15070-063)) was added and gently pipetted up and down and passed through the cell strainer. The cell suspension was centrifuged at 200×*g* for 10 min at room temperature and the supernatant discarded. The cells were re-suspended in 5 ml of cell culture medium (with freshly added 50 ng/ml of M-CSF (BioLegend, 576406)) and seeded in a T25 or T75 flask at a cell density of 5 × 10^6^. After 3–4 days the mixed culture reached confluence, and microglia were loosely attached to the mixed culture layer. To collect microglia the cell culture flask was shaken and the medium was collected into a new falcon tube. Primary microglia were counted and cultured in culture medium supplemented with 50 ng/ml of M-CSF (BioLegend, 576406).

### Isolation and culturing of murine bone-marrow-derived-macrophages

Bone-marrow-derived-macrophages (BMDMs) were isolated from femurs and tibiae of 8–12-week-old WT and KO mice. After dissection, the bones were isolated, cleaned with soft tissue and disinfected with 70% ethanol and put in ice-cold 1× PBS in a 50 ml falcon tube. The bones were cut carefully at the ends, and the bone marrow was flushed out with sterile ice-cold 1× PBS using a 26-gauge needle. During this process, the bone marrow suspension was kept on ice. Then the bone marrow was pelleted down by centrifugation for 10 min at 500×*g*. The supernatant was discarded and the bone marrow mixed in macrophage culture media (1× DMEM + 10% FBS + 100 U/ml Pen/Strep + 50 ng/ml of M-CSF). Bone marrow progenitor cells were counted and plated at a density of 4 × 10^5^ cells in one 10-cm plastic Petri dish with 8 ml of macrophage culture medium. This was marked as day 0 of BMDMs culture.

On day 2, 8 ml of fresh macrophage culture media was added. At day 5, the macrophages were collected after removing and washing debris with ice-cold 1× PBS. The BMDMs were pelleted after centrifugation for 10 min at 500×*g* and were counted. The required number of cells was plated with macrophage culture media in either 10 cm tissue culture plates, 96-well plates, or 6-well plates according to the requirement of experiment. On day 6, the cells were stimulated and harvested for downstream assays.

### Isolation and differentiation of human PBMCs into macrophages

The local ethics committee of Jena University Hospital approved the study (No. 2023- 3050-Material) and a written consent was obtained from all participants. Blood was collected from patients with SPG11 and healthy volunteers from the Jena University Hospital. The blood was carefully layered on top of Lympholyte-H solution (Cedarlane, CL5015-R) and centrifuged at 800×*g* for 20 min. The lymphocyte layer was transferred to a new tube and the cells washed with 1× PBS and centrifuged at 800×*g* for 5 min. The pelleted PBMCs were collected and plated in macrophage culture medium (1× DMEM + 10% FBS + 100 U/ml Pen/Strep + 20 ng/ml of human M-CSF (BioLegend, 574806)). Frozen PBMCs from patients with SPG11 and healthy volunteers were also obtained from the biobank of Tübingen University Hospital. The cells were de-frosted and pelleted down by centrifugation at 800×*g* for 10 min. The supernatant was discarded. Cells were counted and plated in macrophage culture medium (1× DMEM + 10% FBS + 100 U/ml Pen/Strep + 20 ng/ml of human M-CSF) according to the requirements of cellular assays. This was marked as day 0, fresh macrophage culture medium was added on day 2 and cells cultured till day 5 prior to stimulation for downstream assays. The demographic data from patients with SPG11 and healthy controls is provided as Supplementary Table S1.

### *In vitro* stimulations

For the canonical activation of the NLRP3 inflammasome, cells were primed with 200 ng/ml of LPS (from *E. coli* O111:B4, InvivoGen, tlrl-eblps) for 3 h. Cells were seeded at a density of 80,000 cells/well in a 96-well plate or 2.5 million cells/10 cm dish. After priming, the cells were washed twice with Opti-MEM® (Gibco, 31985070) followed by incubation with 5 μM nigericin (tlrl-nig, Invivogen) in Opti-MEM® medium for 3 h.

For the non-canonical activation of the Caspase-11 inflammasome, cells were first primed with 200 ng/ml of LPS (InvivoGen, tlrl-eblps) for 3 h. After priming, the cells were washed twice with Opti-MEM® media and transfected with 500 ng/ml of LPS (InvivoGen, tlrl-eblps) at a final volume of 100 μl/well for 96-well plates and 5 ml/plate for 10 cm plates with 0.5% Lipofectamine™ 2000 (ThermoFisher Scientific, 11668027) in Opti-MEM® media. The cells were incubated for an additional 3 h before being harvested. Controls were carried out by stimulating cells with either LPS alone or with Lipofectamine™ 2000 alone. Cell-free supernatant was harvested from 96-well plates. LDH release and cytokine release were measured by ELISA. Cells stimulated in 10 cm dishes were harvested and analysed by immunoblotting.

### Immunoblot analysis

For protein isolation the cells were lysed with RIPA buffer (50 mM Tris–HCl pH 8.0, 120 mM NaCl, 0.5% NP-40, 0.5% sodium-deoxycholate, 2 mM EDTA) with freshly added cOmplete™ protease inhibitor mix (Roche, 4693116001) and PhosSTOP™ (Roche, 4906845001) on ice. The tubes containing cell suspension were incubated on ice for 10 min, followed by gentle shaking at 4 °C for 30 min and centrifuged at 14,000×*g* for 15 min at 4 °C. The resulting supernatants were collected and protein concentration quantified with the Pierce™ BCA protein assay kit (ThermoFisher, 23225) according to the manufacturer's instructions. The protein samples were adjusted with cell lysis buffer and 4× SDS sample buffer to a concentration of 1–3 μg/μl. Methanol and chloroform were used to precipitate proteins from the media of stimulated cells. 1/4th volume of chloroform and 1 volume of methanol were mixed with the cell-free supernatant, vigorously vortexed for 30 s, and centrifuged at 10,000×*g* for 10 min at room temperature. Precipitated proteins were carefully collected from the middle layer and washed once again with methanol and centrifuged at 10,000×*g* for 5 min at room temperature. The methanol was removed and the pellet dried at 50 °C for approximately 3–5 min. Then, the pellet was re-suspended in 1× Laemmli buffer.

For protein isolation from tissue, tissue lysis buffer (137 mM NaCl, 25 mM Tris, 2.7 mM KCl, 0.1% Triton X-100 (ThermoFisher Scientific, 13444259), pH 7.6, and protease and phosphatase inhibitor cocktail) was added at a ratio of 20% W/V to tissues and homogenised using a tissue potter. After incubation on ice for 15 min, the material was sonicated for 2 min at 4 °C. Cell debris was removed by centrifugation for 20 min at 12,000×*g*, and the supernatant was collected in a new tube. The protein concentration was quantified with the Pierce™ BCA protein assay kit according to the manufacturer's instructions.

The protein samples were analysed via SDS-PAGE and transferred to PVDF membranes (Cytiva, Amersham, 10600023). Membranes were washed for 5 min at room temperature with TBS-T (tris-buffered saline with 1% tween 20, pH 7.4) followed by blocking with 5% milk powder (Santa Cruz biotechnology, sc-2324) or 2–5% BSA (Serva, 11930.03). After 3 washes with TBS-T, 5 min each, membranes were incubated with the respective primary antibodies in TBS-T at the dilution indicated at 4 °C. The next day, membranes were incubated with the respective secondary antibodies in TBS-T at the dilutions indicated for 1 h at room temperature. The Clarity Western ECL Substrate (Biorad, 1705061) was used for the detection (ImageQuant, LAS 4000). Antibodies used for this study are available as Supplementary Table S2. ImageJ was used for measuring the band densitometry.

### *In vitro* bacterial infections

The well characterised bacterial strains *E. coli* (DSM 10724), *S. typhimurium* (ATCC 14028), and *Staphylococcus aureus* (LS-1) were obtained from the bacterial strain collection of Institute of Medical Microbiology, Jena University Hospital. Bacteria were cultured to the logarithmic growth phase, and optical density (OD) 1 suspensions were prepared from these cultures for infection of BMDMs. Cells were seeded at a density of 250,000 cells/well in 6-well plates. The next day, OD1 bacteria were washed 2× in sterile PBS and centrifuged at 5000 rpm for 10 min and re-suspended in the same volume of invasion medium (1× DMEM + 10 mM HEPES (Gibco, 15630080) + 1% Human serum Albumin (PAN-Biotech, 1437317)) to maintain the OD1. For infection, the multiplicity of infection (MOI) was 10, cells were washed twice with sterile 1× PBS and media were replaced by the invasion medium with the respective bacterial strain. Cells were placed in a humidified incubator (5% CO2, 37 °C) for 90 min, then extra-cellular bacteria were killed with incubation of the cells with stop medium (1× Opti-MEM® + 200 μg/ml Gentamycin (ThermoFisher Scientific, 15750060)). After 3 h, the supernatants were collected and the released cytokines measured.

### ELISA

The supernatants from inflammasome-activated primary microglia, BMDMs, human MDMs, bacterial infected BMDMs, and blood serum of LPS-treated mice were used to measure cytokines by ELISA (Supplementary Table S3) according to the manufacturer's protocol. Absorbance was detected with a Tecan Infinite® 200 pro plate reader. Cytokines in the supernatant were calculated using data points generated from the standards provided with ELISA kits.

### Lactate dehydrogenase (LDH) assay

The LDH-Cytox™ assay kit (Supplementary Table S3) was used according to the manufacturer's protocol. Cells were removed from the culture medium by centrifugation at 500×*g* at 4 °C for 5 min prior to LDH release determination. 50 μl of supernatant from each well were transferred into a new 96-well plate and 50 μl of the reaction mixture of the catalyst and tetrazolium salt (1:50) was immediately prepared and mixed with the supernatant. The absorption was detected at 490 nm with the Tecan Infinite® 200 pro plate reader.

### Real-time fluorescence permeability assay

Cell permeability of BMDMs was measured in real-time after the canonical activation of the inflammasome using propidium iodide (PI) uptake. After 3 h priming with LPS, cells were washed with PBS and PI (1 μg/ml, Sigma) was added in combination either with nigericin (5 μM) or without nigericin in Opti-MEM® media. Cells were incubated for 10 min in a humidified incubator (37 °C and 5% CO_2_) prior to take readings at the plate reader. Real-time fluorescence monitoring was carried out using a Tecan plate reader. The excitation wavelength was set to 530 nm with a bandwidth of 9 nm, and the emission wavelength was set to 617 nm with a bandwidth of 20 nm. The absolute gain was determined by calculating the signal from 1% Triton-X-100 lysed cells. The temperature was maintained at 37 °C throughout the assay.

### Immunofluorescence assays

For immunofluorescence studies, cells were seeded on sterile coverslips in a 24-well-plate. After treatments, cells were fixed with chilled 100% methanol on ice for 10 min. For intracellular staining, cells were permeabilised for 15 min with saponin-based permeabilisation buffer (Invitrogen) in PBS. Then, cells were blocked with 5% normal-goat-serum (BIOZOL, VEC-S-1000) in 1× PBS for 1 h at room temperature and incubated with primary antibody (Supplementary Table S2) overnight in a humidity chamber at 4 °C with gentle agitation. The next day, cells were washed thoroughly with 1× PBS and stained with the respective secondary antibody (Supplementary Table S2) for 1 h at room temperature under gentle agitation in the dark. After washing, cell nuclei were stained with DAPI, washed and mounted. The slides were dried overnight at RT in the dark and kept at 4 °C until analysis. Images were taken with either a 40× objective of a Keyence BZ-X microscope or with a confocal scanning fluorescence microscope (Zeiss LSM 880) with Airyscan using the Plan-Apochromat 63×/1.4 oil DIC M27 objective and further analysed with ImageJ.

### RNA isolation

The cells were washed with cold 1× PBS, then lysed in 500 μl TRIzol™ Reagent (Thermo Fisher Scientific, 15596026) for each well of 6-well cell culture plates. RNA was isolated by chloroform/phenol extraction. After washing with 75% ethanol, the RNA was dissolved in nuclease-free water and the concentration determined using the NanoDrop device.

### Quantitative real-time PCR

For quantitative real-time experiments, cDNA was synthesised from SuperScript™ III reverse transcription kit (ThermoFisher, 18080-044) according to the manufacturer's instructions. For single reactions, the PowerUp SYBR® Green Master mix (Applied Biosystems, A25742) was combined with specific primers (200 pmol) and 5–20 ng cDNA. Reactions and negative controls were performed in technical triplets. Data analysis was done using Bio-Rad software (CFX Maestro). Primers used for this study are available as Supplementary Table S4.

### Mass spectrometry sample preparation

To isolate proteins from BMDMs, 5 million cells were homogenised in 2 ml of cold hypotonic buffer (250 mM Sucrose, 3M KCl, 1 M MgAc, 100 mM DTT, 1 M HEPES pH 7, 1 mM CaCl_2_, 1 mM MgCl_2,_ and protease inhibitor cocktail). Cells were homogenised using at least 50 strokes and passed through a 21-G syringe for at least 10 times. Nuclear fragments were separated by centrifugation at 500×*g* for 5 min at 4 °C, then cytosol soluble protein fractions and organelle fraction were separated by centrifugation at 20,000×*g* for 30 min at 4 °C. The supernatant containing the cytosol soluble protein fraction was carefully shifted to new tubes and the pelleted organelle fraction was mixed with 500 μl of hypotonic solution. The protein concentrations were quantified with the Pierce™ BCA protein assay kit according to the manufacturer's instructions and samples stored at −80 °C till further processing. For each cytosolic or organelle fraction, 100 μg of protein were transferred to fresh microtubes and filled up to 90 μl with 4% SDS 50 mM triethylammonium bicarbonate (TEAB) buffer, pH 8.0. Proteins were reduced by addition of 2 μl 1 M DTT and incubation at 55 °C for 30 min, in a thermomixer (600 rpm), followed by alkylation with 2 μl 2 M acrylamide^30^ and incubation for 30 min at room temperature (RT), 600 rpm. The reaction was quenched by addition of 2 μl 1 M DTT and incubation for 15 min at RT, 600 rpm. Samples were then subjected to SP3-digestion, as described previously.^31^ Briefly, 10 μl of a pre-washed SP3-bead mixture was added to each sample, mixed by pipetting, and protein-to-bead binding was induced by the addition of 3× sample volumes of 96% ethanol and incubation for 5 min at 24 °C, in a thermomixer (1000 rpm). Using a magnet, supernatants were removed and SP3-beads washed 3× with 360 μl 80% ethanol. The bead-bound proteins were digested with trypsin at an enzyme to sample ratio of 1:25 (w/w) in 200 μl 50 mM TEAB pH 8.0 at 37 °C, for 14 h in a thermomixer (1000 rpm). Finally, SP3-beads were collected on a magnet, supernatants containing the peptides transferred to fresh microtubes, dried in a vacuum centrifuge, and stored at −80 °C. Peptide yields were determined using the Pierce Quantitative Fluorometric Peptide Assay (ThermoFisher Scientific, Waltham, MA).

### LC-MS analysis

Samples were analysed using an UltiMate 3000 RSLCnano UHPLC connected to an Orbitrap Fusion Lumos mass spectrometer (both ThermoFisher Scientific). Peptides were separated on an in-house prepared capillary emitter column (length: 40 cm, OD: 360 μm, ID: 100 μm) packed with reversed phase material (3 μm Reprosil AQ C_18_, Dr. Maisch, Germany). For each sample, 1 μg of peptides were loaded at a flow rate of 850 nl/min 98% buffer A (0.1% [v/v] formic acid) 2% buffer B (90% [v/v] acetonitrile, 0.1% [v/v] formic acid) directly onto the analytical column and separated with a 120 min linear gradient from 2% to 35% buffer B at a flow rate of 300 nl/min. The mass spectrometer was operated in the positive ion mode. For data-independent acquisition (DIA) analyses, MS1 spectra were acquired every 3 s from *m*/*z* 350–1200 in the Orbitrap at a resolution of 120,000, a normalised automated gain control (AGC) target setting of 125% and a maximum injection time of 20 ms. Peptides ions were isolated in the quadrupole in 36 individual 24 *m*/*z* windows covering the mass range of the MS1 scan and fragmented in the higher-energy collisional dissociation (HCD) fragmentation mode at a normalised collision energy (NCE) of 30. Fragment ion spectra were measured in the Orbitrap from *m*/*z* 200–2000 at a resolution of 30,000, a normalised AGC target setting of 2000% and a maximum injection time of 60 ms.

Moreover, four sample pools comprising equal amounts of samples from the same sample type and treatment (cytosolic/20,000×*g* pellet and base/iLPS) were separated by a 240 min linear gradient in the data-dependent acquisition (DDA) mode for library generation. MS1 spectra were acquired every 5 s at *m*/*z* 350–1200 *m*/*z* and a resolution of 120,000 (normalised AGC target setting: 100%, maximum injection time: 50 ms). The most intense precursors (intensity threshold: 2 × 10^4^, charge states: 2–6+) were fragmented by HCD (isolation width: 1.6 *m*/*z*, NCE: 30%) in the top speed mode (cycle time: 3 s) with dynamic exclusion enabled (duration: 60 s). Fragment ion spectra were recorded in the Orbitrap at a resolution of 30,000 with an automatic scan range, a normalised AGC target setting of 100%, and a maximum injection time of 54 ms).

### Mass spectrometry data analysis

For cytosolic and 20.000×*g* fractions separate spectral hybrid libraries were generated from DIA/DDA data with Spectronaut (version 16.0.220606.53, Biognosys, Switzerland) in combination with SwissProt mouse (release 2022_01_07, 17,090 entries) and a common contaminants database^32^ (381 entries). Trypsin/P was selected as enzyme with 2 allowed missed cleavage sites and the peptide length was restricted to 7–52 amino acids. Propionamide (C) was set as fixed modification and acetyl (protein N-term) and oxidation (M) as variable modifications. DIA files were matched against the respective library and the following settings were used for peptide/protein identification/quantification: XIC extraction window: “maximum intensity”; mass tolerance and iRT calibration: “automatic”; precursor/protein q-value (experiment): 0.01; protein q-value (run): 0.05; precursor PEP cutoff: 0.2; quantification precursor filtering: “identified (q-value)”; quantity MS level: “MS2”; protein LFQ method: “automatic”; imputation (“background signal”) and cross-run normalisation (“automatic”): enabled. Differentially expressed proteins were determined in an unpaired Welch t-test using both MS1 and MS2-level information and data exported to MS Excel for further processing. The mass spectrometry proteomics data have been deposited to the ProteomeXchange Consortium via the PRIDE^33^ partner repository and are publicly available (https://proteomecentral.proteomexchange.org/ui) with the dataset identifier PXD065699. Gene ontology (GO) enrichment analysis was performed with online OMICs tool www.metascape.org.^34^ Regulated proteins which exhibited an absolute log 2 fold change of 0.58 (1.5-fold up or down) and had a q-value <0.05 (Benjamini-Hochberg FDR assessment of p-values) were used for GO analysis.

### LPS-induced inflammation in mice

8-12-week-old WT and *Spg11* KO mice were used for the experiment. On experiment day (day 0), two different doses (5 or 20 mg/kg of body weight) of LPS (from *E. coli* O111:B4. InvivoGen, tlrl-eblps) were administered intra-peritoneally in 5 μl/g body weight 0.9% NaCl (saline). A control group (5 mice per genotype) received 5 μl/g body weight saline (without LPS). From two cohorts which received a high dose of LPS (20 mg/kg of body weight), the 1st cohort (9 mice per genotype) was analysed for survival analysis. The 2nd cohort (8 mice per genotype) was used to collect blood after 4 h and 24 h of LPS/saline injection. Approximately 70–120 μl of blood was taken by retro-bulbar venous plexus puncture under isoflurane anaesthesia (induction 2–3% with approximately 0.1–0.2 ml of O_2_/min). Mice were sacrificed to collect blood and organs at 24 h.

The animals (5 mice per genotype) which received a low-dose of LPS (5 mg/kg of body weight) were weighed at the time of injection and every 12 h, then they were phenotypically examined for their clinical score (Supplementary Table S5) every 4–8 h. This cohort was sacrificed after 96 h of LPS/saline injection, under isoflurane anaesthesia (induction 2–3% with approximately 0.1–0.2 ml of O_2_/min), and organs were collected for further analysis for inflammation.

### Cryo-sectioning and immuno-histochemistry

Mice were anaesthetised with a mixture of Ketamine (100 mg/kg body weight) and Xylazine (16 mg/kg body weight) and perfused transcardially with 4% PFA in 1xPBS. Brains were dissected after perfusion and cryo-protected with increasing concentrations of sucrose, 10% and 30% in 1× PBS, for 6 h and overnight respectively. 40 μm sections were cut with a sliding microtome (Leica SM 200R) and floating sections were stored in 1× PBS supplemented with 0.025% NaN_3_. For IBA-1 staining of free-floating tissue sections, sections were washed three times for 10 min each, followed by a final wash for 10 min in 0.25% Triton X-100/1xPBS on gentle agitation. Next, the tissue was incubated in blocking buffer (5% normal-goat-serum/1xPBS) for 1 h at room temperature (RT) and the primary antibody, diluted in 5% normal-goat-serum, was added and incubated overnight at 4 °C while gently shaking. The next day, the sections were washed three times and the appropriate secondary antibody added, diluted in 5% normal-goat-serum/1xPBS, and incubated for 1 h at RT. After washing, the nuclei were stained with DAPI (1:10,000) in 1× PBS for 10 min. After final washes with 1× PBS sections were transferred onto Superfrost® microscope slides (Roth, 1879.1) and mounted with Flouromount-G™ (Invitrogen, 00-4958-02) and a coverslip. Brains from LPS-induced mice were preserved in Tissue-Tek® O.C.T. compound (Sakura), cut on cryostat (CryoStar™ NX70), mounted on HistoBond® glass slides (Roth, CEX0.1) and stored at −80 °C. For staining, the slides were thawed on RT and then 4% PFA was directly put on the tissue for 20 min at 4 °C in a humid chamber. Three washing steps, 5 min each were done with 1× PBS, and slides transferred to Shandon™ chambers. The tissue was permeabilised with PBS-T for 30 min, followed by two 10 min washing steps with 1× PBS. Then slides were incubated with 5% NGS in PBS-T for 1 h at RT. The primary antibody (IBA-1) was then diluted in the blocking solution and incubated with the tissue overnight at 4 °C. The next day, slides were washed three times with 1× PBS and incubated with the appropriate secondary antibody for 1 h at RT. Then nuclei were stained with DAPI for 10 min at RT. Finally, after washing, the slides were mounted with Flouromount-G™ and left overnight in the dark at RT. The images were taken using a confocal microscope (LSM 880 ZEISS) using a 20× objective. IBA-1 positive structures were counted using the cell counter plugin of FIJI ImageJ and the values were normalised to the total area of the brain tissue in the respective frame.

### Statistical analysis, online resources and softwares

The GraphPad Prism 8 software (GraphPad Software, Inc.) was used for statistical analyses and data representation. The appropriate statistical tests, such as two-tailed Student's t-test, Log–Rank test, Mann–Whitney U test, two-way ANOVA etc., were performed as indicated in the figure legends. Data from the experiments are represented as mean ± SEM. GO enrichment analysis were performed with online OMICs tool (http://www.metascape.org). Few images are made with BioRender.com (https://biorender.com/8ocwxff, https://biorender.com/7fizpzf).

### Role of funders

The funding agencies neither had a role in the design of this study, nor the collection, analysis, and interpretation of the data, nor the writing and the decision to submit the manuscript for publication.

## Results

### Neurodegeneration in *Spg11* KO mice leads to the activation of microglia

**Fig. 1:**
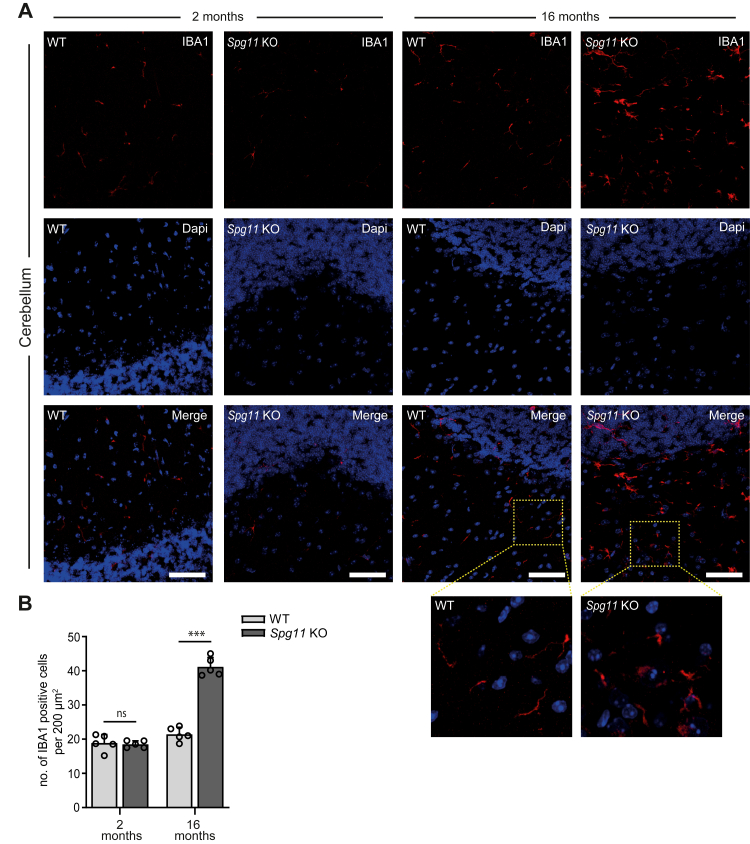
**Increased numbers of IBA1-positive cells in brains of 16- but not in 2-month-old *Spg11* KO mice. A,** Brain sections of 2- and 16-month-old WT and *Spg11* KO mice stained for IBA1 (red) and DAPI (blue). Scale bar, 50 μm. Insets show higher magnifications. **B,** Quantification of IBA1-positive cells in WT and S*pg11* KO mice at 2 and 16 months of age (n = 25 brain sections from 5 mice per genotype; two-way ANOVA followed by Sidak's multiple comparison test: ∗∗∗p < 0.001, ns-not significant). Quantitative data are shown as mean ± SEM.

We previously reported that *Spg11* KO mice develop a progressive gait disorder starting around 8 months of age, which is preceded by the progressive intracellular accumulation of autophagic material and neuron loss.^11^ In agreement with published data,^13^ we found a strong increase in IBA1 (Ionised calcium-binding adaptor molecule 1)-positive microglia in 16-month-old KO mice compared to controls (Fig. 1A and B). IBA1 is primarily expressed in activated microglia and performs membrane ruffling and phagocytosis.^35^ At 2 months of age, when KO mice are still asymptomatic and do not yet show neuron loss,^11^ the number of IBA1-reactive microglia did not differ between genotypes.

Thus, the activation of microglia in SPG11 likely occurs secondary to neurodegeneration in mice.

### The increase in IBA1-positive cells in response to intraperitoneal injection of LPS is enhanced in presymptomatic *Spg11* KO mice brain

**Fig. 2:**
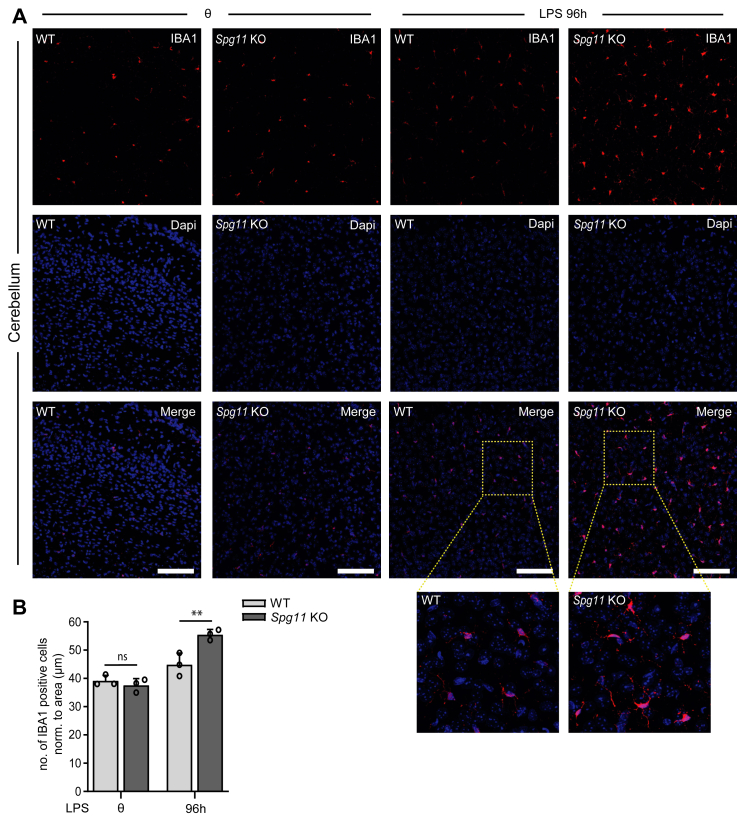
**Intraperitoneal injection of LPS at 2 months of age activates microglia more strongly in *Spg11* KO mice. A,** IBA1-staining of brain sections from 2-month-old WT and *Spg11* KO mice sacrificed 96 h after intraperitoneal injection of *E. coli* LPS (5 mg/kg body weight). Scale bar, 50 μm. Insets show higher magnifications. **B,** Quantification of IBA1-positive-cells in WT and *Spg11* KO mice (n = 15 brain sections from 3 mice per genotype; two-way ANOVA followed by Sidak's multiple comparison test: ∗∗p < 0.01, ns-not significant). Quantitative data are shown as mean ± SEM.

Since Spatacsin is broadly expressed including microglia,^36^^,^^37^ we wondered whether the disruption of *Spg11* may affect microglia in a cell-autonomous manner, thus possibly sensitising microglia to neuro-inflammatory stimuli. To induce neuro-inflammation *in vivo*, we injected LPS, a strong pro-inflammatory agent, into the peritoneal cavity of 2-month-old WT and *Spg11* KO mice (5 mg/kg body weight). Four days later, we sacrificed the mice and quantified IBA1-positive reactive microglia in brain sections. Notably, the number of IBA1-positive microglia increased in response to systemic LPS-administration in both genotypes, however, the increase was more pronounced in the KO cohort (Fig. 2A and B).

These data suggest that Spatacsin loss-of-function may indeed have cell-intrinsic consequences in murine microglia.

### Disruption of Spatacsin selectively augments the non-canonical but not the canonical NLRP3 inflammasome in primary microglia

LPS can trigger the assembly of large cytosolic multiprotein complexes, also known as inflammasomes, which are crucial for the activation of inflammatory caspases and the subsequent processing and release of pro-inflammatory mediators.^38^^,^^39^ The activation of the inflammasome requires both priming and activation signals. Priming is mediated primarily by triggering PRRs such as toll-like receptors (TLR) e.g. via extracellular LPS, which drives the expression of pro-IL-1β, NLRP3, and Caspase-11.^40^ In the second step, DAMPs such as ATP, PAMPs, or nigericin activate Caspase-1 and thus IL-1β processing.^39^^,^^41^ Caspase-dependent cleavage of pro-GSDMD and oligomerisation of mature GSDMD (p30) in the plasma membrane leads to the formation of membrane pores, which facilitate the release of IL-1 family cytokines and induce pyroptosis.^18^^,^^42^, ^43^, ^44^ In contrast, the non-canonical pathway is activated by intracellular LPS (iLPS) via different paths such as phagocytosis and vesicle-mediated delivery^45^^,^^46^ and leads to the activation of Caspase-11 in mice or Caspases-4 and -5 in humans resulting in cleavage of N-terminal GSDMD and pyroptosis, in an Caspase-1-independent manner.^39^^,^^47^^,^^48^

**Fig. 3:**
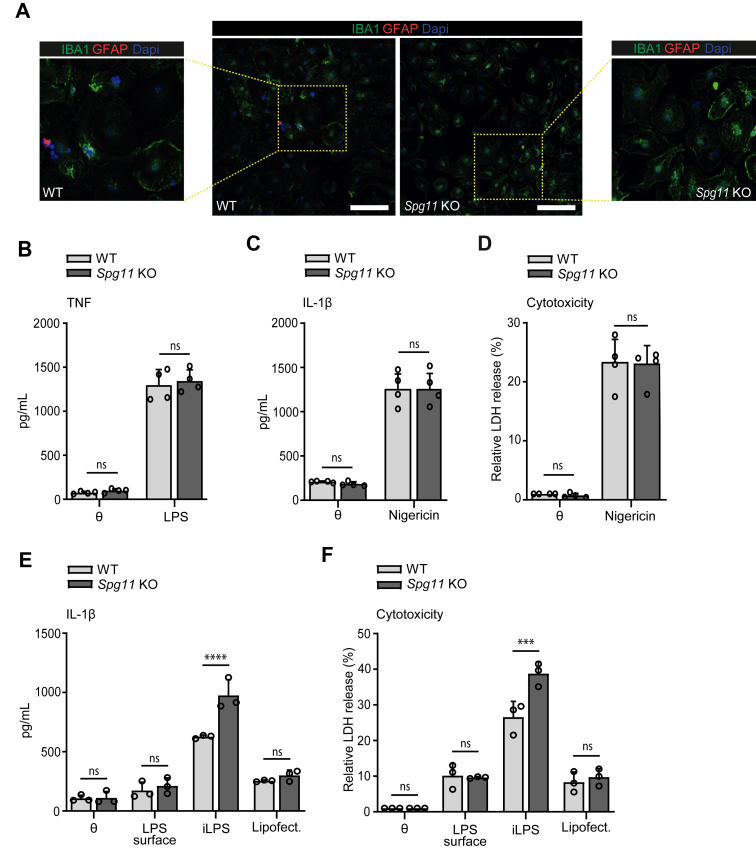
**Spatacsin loss-of-function sensitises the non-canonical but not the canonical inflammasome in primary microglia. A,** Primary microglia cultures co-stained for IBA1 (green) to label microglia and GFAP (red) to label astroglia. Scale bar, 10 μm. Insets show higher magnifications. **B,** TNF release assessed by ELISA (n = 4 independent experiments; two-way ANOVA followed by Sidak's multiple comparison test: ns-not significant). **C, D,** Primary microglia were primed with LPS (200 ng/ml) for 3 h, before they were treated with nigericin (5 μM) for 3 h to activate the canonical inflammasome. Both IL-1β release (C) and relative LDH release (D) into cell culture supernatants were measured after activation of the canonical pathway for both genotypes (n = 4 independent experiments; two-way ANOVA followed by Sidak's multiple comparison test: ns-not significant). **E, F,** To activate the non-canonical inflammasome, we applied 500 ng/ml intracellular LPS (iLPS) using Lipofectamine (iLPS for 3 h) in LPS-primed microglia. IL-1β and LDH release from WT and *Spg11* KO microglia were measured (n = 3 independent experiments; two-way ANOVA followed by Sidak's multiple comparison test: ∗∗∗∗p < 0.0001, ∗∗∗p < 0.001, ns-not significant). Quantitative data are shown as mean ± SEM.

To get further insights into the role of Spatacsin for microglia activation, we isolated a pure population of primary microglia from new-born (P2–P4) WT and KO mice. Cells from both genotypes did not show any structural abnormalities (Fig. 3A). Microglia from WT and KO mice were primed with LPS (200 ng/ml) for 3 h and both genotypes showed comparable TNF accumulation in the supernatant in response to LPS stimulation (Fig. 3B), suggesting that disruption of Spatacsin does not affect the priming phase. Following LPS priming, the microglia were stimulated with nigericin, a specific activator of the NLRP3 inflammasome.^41^^,^^49^ Notably, IL-1β release and lactate dehydrogenase (LDH) release in the supernatant as a hallmark of inflammasome activation and pyroptosis increased but did not differ between both genotypes (Fig. 3C and D) suggesting that the canonical inflammasome is independent of Spatacsin.

To activate specifically the non-canonical inflammasome we used lipofectamine to deliver LPS intracellularly (iLPS) after priming.^50^ While LPS or lipofectamine alone had no effect, their combination evoked the release of IL-1β, which was strongly enhanced for *Spg11* KO microglia (Fig. 3E) as was LDH release (Fig. 3F).

These data show that the disruption of Spatacsin sensitises the activation of the non-canonical, but not the canonical inflammasome in murine microglia.

### Disruption of Spatacsin also sensitises the non-canonical inflammasome in bone-marrow-derived-macrophages

**Fig. 4:**
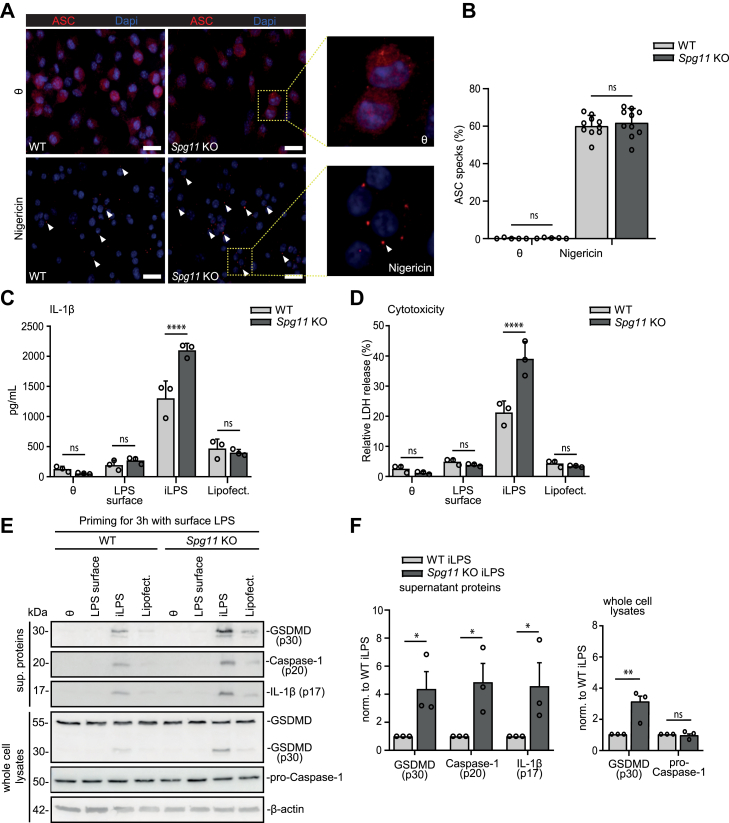
**Disruption of Spatacsin specifically promotes the activity of the non-canonical inflammasome in BMDMs. A,** ASC staining of WT and *Spg11* KO BMDMs at steady state and after nigericin activation. Primary BMDMs were primed with 200 ng/ml extracellular LPS for 3 h before applying 5 μM nigericin for 3 h. Some oligomerised ASC specks are marked by white arrows. Scale bar, 10 μm. Insets show higher magnifications. **B,** Quantification of ASC speck-positive cells for genotypes (n = 10 images/condition from 3 independent experiments; Student's t-test: ns-not significant). **C, D,** The release of IL-1β (C) and LDH (D) from LPS-primed WT and *Spg11* KO BMDMs was measured after activation of the non-canonical pathway with iLPS (n = 3 independent experiments; two-way ANOVA followed by Sidak's multiple comparison test: ∗∗∗∗p < 0.0001, ns-not significant). **E, F,** Immunoblot analysis of cell culture supernatants and total cell lysates of iLPS-activated WT and *Spg11* KO LPS-primed BMDMs for different markers of inflammasome activity. The abundances of GSDMD (p30), Caspase-1 (p20), and IL-1β (p17) were determined in cell culture supernatants. GSDMD (p30) and pro-Caspase-1 were also measured in cell lysates (n = 3 independent experiments; two-sided paired Student's t-test: ∗p < 0.05, ∗∗p < 0.01, ns-not significant). Quantitative data are shown as mean ± SEM.

Macrophages are closely related to microglia. Both guard their territories, phagocytose pathogens and other offenders, and secrete an array of pro- and anti-inflammatory cytokines and growth factors. Therefore, we aimed to analyse whether the disruption of Spatacsin also sensitises macrophages. To study macrophages *in vitro* we differentiated bone marrow progenitor cells to form BMDMs from WT and *Spg11* KO mice. BMDMs were primed with extracellular LPS (200 ng/ml) for 3 h. Suggesting that the priming of BMDMs is not affected by the disruption of Spatacsin, the expression of pro-Caspase-11, pro-IL-1β, or GSDMD (Supplementary Fig. S1A, B), the transcript abundance for *Caspase-11* (Supplementary Fig. S1C), and levels of TNF (Supplementary Fig. S1D) did not differ between genotypes. As in microglia, this suggests that the priming of BMDMs is not affected by the disruption of Spatacsin. Activation of the canonical inflammasome with nigericin also elicited a comparable number of large complexes of apoptosis-associated speck-like protein containing a caspase recruitment domain (ASC), called specks, which associated with NLRP3 to recruit Caspase-1^51^ irrespective of the genotype (Fig. 4A and B). In accordance, the abundance of pro-Caspase-1 and cleaved GSDMD (p30) in cell lysates and Caspase-1 (p20), cleaved GSDMD (p30) and IL-1β (p17) in supernatants did not differ between genotypes (Supplementary Fig. S1E–G). This was also true for pyroptosis, which was assessed by propidium iodide (PI) uptake (Supplementary Fig. S1H).

*S. aureus,* a gram positive bacteria, is known for the canonical inflammasome activation.^52^ When we infected WT and *Spg11* KO BMDMs with *S. aureus*, the release of IL-1α, IL-1β and TNF did not differ between genotypes upon infection with *S. aureus* (Supplementary Fig. S2A), thus confirming that the canonical inflammasome activation is not affected.

We next assessed the response to iLPS (500 ng/ml) in BMDMs, which triggered a much stronger release of IL-1β and LDH (Fig. 4C and D). This was accompanied by an increased abundance of Caspase-1 (p20), IL-1β (p17) as well as cleaved-GSDMD (p30) in the supernatant and protein lysates of *Spg11* KO BMDMs, while the abundance of pro-Caspase-1 did not differ between genotypes (Fig. 4E and F). *E. coli* and *S. typhimurium*, are known to activate the non-canonical inflammasome due to LPS as an integral component.^17^^,^^45^ In agreement with our findings for iLPS, *Spg11* KO BMDMs infected with Gram-negative *E. coli* or *S. typhimurium* specifically secreted more IL-1α and IL-1β but no change was observed for TNF release between genotypes (Supplementary Fig. S2B, C).

These data show that the disruption of Spatacsin sensitises the activation of the non-canonical, but not the canonical inflammasome.

### The non-canonical inflammasome is hyper-responsive in monocyte-derived-macrophages from patients with SPG11

**Fig. 5:**
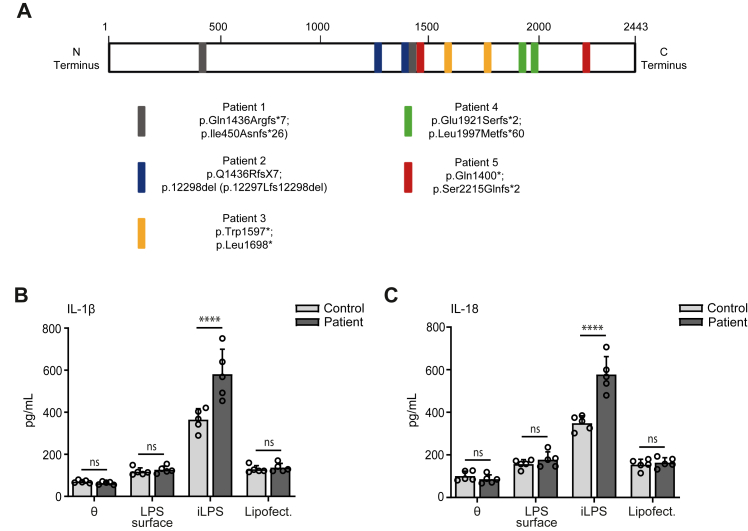
**The non-canonical inflammasome is hyperactive in MDMs isolated from patients with SPG11. A,** Schematic representation of full length Spatacsin (2443 amino acids)^54^ and the respective localisation of the variants identified in patients (shown as coloured boxes). **B, C,** Peripheral blood monocytes from patients with SPG11 and controls were differentiated into macrophages. After priming with LPS (200 ng/ml for 3 h) and activation of the non-canonical pathway with iLPS (500 ng/ml for 3 h), the levels of the effector cytokines IL-1β (B) and IL-18 (C) were measured for patients with SPG11 and healthy controls (n = 5 patients and controls each; two-way ANOVA followed by Sidak's multiple comparison test: ∗∗∗∗p < 0.0001, ns-not significant). Quantitative data are shown as mean ± SEM.

To test whether these findings also apply for humans, we obtained peripheral mononuclear blood cells (PBMCs) from 5 patients with *SPG11* loss-of-function mutations and 5 age- and sex-matched healthy control donors. One patient (patient-3) had already been reported previously.^53^ All variants of patients included in this study are predicted to result in a loss-of-function of the protein (Fig. 5A). PBMCs were differentiated into human monocyte-derived-macrophages (MDMs). After LPS priming for 3 h, 500 ng/ml of LPS was transfected into the cells. The cells were incubated for additional 3 h and the concentrations of cytokines released into the supernatant were determined by ELISA. Again, we observed a significant increase in the release of IL-1β and IL-18 from MDMs derived from patients with SPG11 as compared to healthy donors (Fig. 5B and C) suggesting a hyper-responsiveness of the non-canonical inflammasome.

Overall, we put on record that disruption of Spatacsin results in the hyper-activation of the non-canonical inflammasome in MDMs in humans.

### Disruption of the Ap5 subunit Ap5z1 also enhances the response of the non-canonical inflammasome in BMDMs

**Fig. 6:**
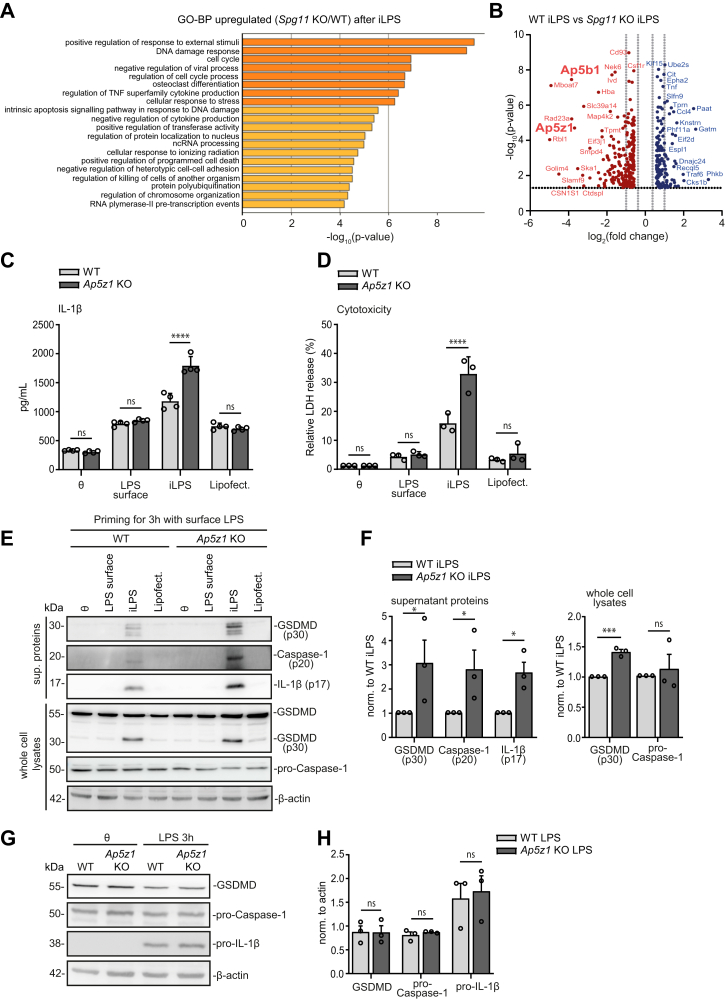
**Disruption of AP5 sensitises the non-canonical but not the canonical inflammasome. A, B,** After activation of primary BMDMs from WT and *Spg11* KO mice (extracellular LPS priming for 3 h and iLPS for 3 h) the proteome was analysed by mass spectrometry. GO-term analysis of the most strongly upregulated biological pathways in activated *Spg11* KO BMDMs (A). Volcano plot of significantly regulated proteins in *Spg11* KO BMDMs (n = 3 mice per genotype) (B). Proteins are deemed regulated if they exhibit an absolute log_2_ fold change and have a q-value less than 0.05 (Benjamini-Hochberg FDR assessment of p-values). **C, D,** IL-1β (C) and LDH (D) release were measured in LPS-primed and iLPS activated *Ap5z1* KO BMDMs (n = 3–4 independent experiments; two-way ANOVA followed by Sidak's multiple comparison test: ∗∗∗∗p < 0.0001, ns-not significant). **E, F,** Immunoblot analysis (E) and quantification (F) of different markers of inflammasome activation in BMDMs protein lysates and supernatants at steady-state and upon activation. Pro-Caspase-1 and GSDMD (p30) levels were measured in cell lysates from WT and *Ap5z1* KO BMDMs. GSDMD (p30), Caspase-1 (p20) and IL-1β were also determined in supernatants from WT and *Ap5z1* KO cells (n = 3 independent experiments; two-sided paired Student's t-test; ∗∗∗p < 0.001, ∗p < 0.05, ns-not significant). **G, H,** GSDMD, pro-Caspase-1, and pro-IL-1β abundances were measured for both genotypes by immunoblot analyses of LPS-primed BMDMs protein lysates (n = 3 independent experiments; two-sided paired Student's t-test: ns-not significant). Quantitative data are shown as mean ± SEM.

To get further insights about the role of Spatacsin in the regulation of the non-canonical inflammasome, we compared the proteome of LPS-primed and iLPS-activated BMDMs from WT and *Spg11* KO mice. In agreement with the sensitisation of the non-canonical inflammasome, stress response regulation and cytokine production were among the most upregulated pathways in KO samples (Fig. 6A). Notably, Ap5z1 and Ap5b1, two subunits of the Ap5 complex, were drastically reduced in KO samples (Fig. 6B). To assess whether AP5 is involved in the regulation of the non-canonical inflammasome, we also assessed BMDMs from *Ap5z1* KO mice.^8^ Indeed, after priming with LPS and subsequent transfection of iLPS, *Ap5z1* KO BMDMs released more IL-1β and LDH compared to controls (Fig. 6C and D). Further supporting a hyper-responsiveness of the non-canonical inflammasome, the abundance of cleaved GSDMD (p30) was increased both in cell lysates and in the supernatants, while pro-Caspase-1 levels did not differ between genotypes (Fig. 6E and F). Caspase-1 (p20) and IL-1β (p17) were also increased in the cell supernatant (Fig. 6E and F). Since the expression of GSDMD, pro-Caspase-1, or pro-IL-1β did not differ between genotypes after LPS priming, we conclude that the disruption of Ap5z1 does not affect the priming signal of BMDMs (Fig. 6G and H).

As evidenced by similar abundances of GSDMD (p30), pro-Caspase-1, pro-IL-1β, and ASC in cell lysates (Supplementary Fig. S3 A, C) as well as cleaved GSDMD (p30), cleaved Caspase-1 (p20), IL-1β and LDH upon activation with nigericin in WT and *Spg48* KO cell supernatants (Supplementary Fig. S3A–E), the canonical pathway is independent of AP5.

Altogether, we conclude that AP5 is involved in the regulation of the non-canonical inflammasome, while the canonical activation is independent of AP5 in mice.

### LPS-induced mortality is increased in *Spg11* KO mice

**Fig. 7:**
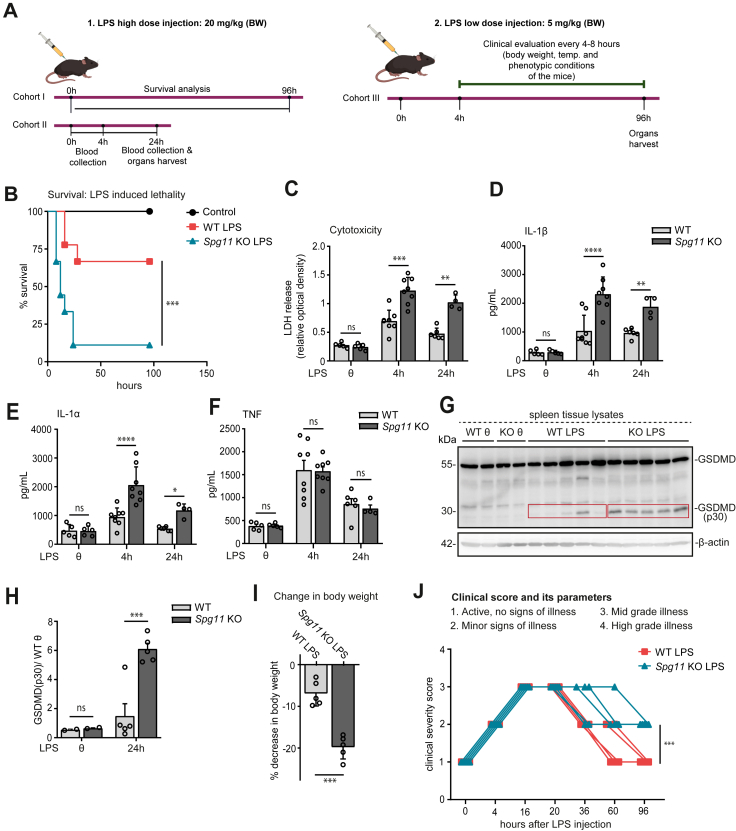
**LPS-induced mortality is increased in *Spg11* KO mice. A,** Mice were challenged by intra-peritoneal injection of either a high dose (20 mg/kg body weight) (1) or a low-dose (5 mg/kg body weight) (2) of LPS. **B,** Survival curve for WT and *Spg11* KO mice after intraperitoneal injection of the high dose of LPS (n = 9 mice per genotype; Log–Rank test: ∗∗∗p < 0.001). **C–F,** LDH (C), IL-1β (D), IL-1α (E), and TNF (F) were measured in blood plasma of WT and *Spg11* KO mice, 24 h after injection of the high dose (n = 4–8 mice per genotype; two-way ANOVA followed by Sidak's multiple comparison test: ∗∗∗∗p < 0.0001, ∗∗∗p < 0.001, ∗p < 0.05, ns-not significant). **G, H,** The abundance of GSDMD (p30) was measured in spleen protein lysates of endotoxemic WT and *Spg11* KO mice (n = 5 mice per genotype; two-way ANOVA followed by Sidak's multiple comparison test: ∗∗∗p < 0.001, ns-not significant). **I,** Body weights after injection of the low dose of LPS between genotypes (n = 5 mice per genotype; two-sided unpaired Student's t-test: ∗∗∗p < 0.001). **J,** Clinical severity score of WT and *Spg11* KO mice (n = 5 mice per genotype; two-way ANOVA followed by Bonferroni post hoc test: ∗∗∗p < 0.001). Quantitative data are shown as mean ± SEM.

Because of the pro-inflammatory phenotype of *Spg11* KO microglia and BMDMs, we wondered whether our findings are relevant *in vivo*. To this end, we challenged asymptomatic 8 to 10-week-old WT and *Spg11* KO mice by intraperitoneal injection of either a high (20 mg/kg bw) or a low (5 mg/kg bw) dose of LPS (Fig. 7A).^55^ After the high dose, the mortality was drastically increased in KO mice: Only 11% of the KO mice survived as compared to 67% for the WT cohort (Fig. 7B). In agreement with a more severe inflammatory induced mortality,^17^^,^^56^ LDH, IL-1β and IL-1α levels were increased in blood serum samples of *Spg11* KO mice (Fig. 7C–E). TNF release, however, did not differ between genotypes (Fig. 7F) indicating that the activation of the TLR4-dependent down-stream signalling was not altered. We also analysed the abundance of cleaved GSDMD (p30) in spleen tissues lysates, which was increased in KO mice (Fig. 7G and H). To avoid high mortality, we next used a low-dose of LPS (5 mg/kg body weight), which allowed us to observe clinical parameters for up to 5 days after the injection in mice. Here, in comparison to WT, *Spg11* KO mice had a more severe body-weight loss (Fig. 7I) and a worse clinical severity score (Fig. 7J)^57^ in line with a more severe inflammatory response.

Taken together, these data suggest that disruption of Spatacsin modulates the activation of the non-canonical inflammasome thus promoting LPS-induced lethality in mice.

## Discussion

Only sporadically diseased patients with SPG11 were examined neuropathologically. From the few reports available, a pronounced infiltration by activated microglia appears to be a consistent finding.^13^^,^^15^ Microglia are the innate immune cells of the brain, which sense and screen surrounding inflammatory cues such as DAMPs and PAMPs like e.g. LPS from Gram-negative bacteria and phagocytose and remove cellular debris.^58^ Microglia activation is observed in various neurodegenerative diseases and can either be protective by phagocytosing and clearing pathological material but also harmful by triggering neuroinflammation and neurodegeneration.^59^ Given that microglia express Spatacsin,^36^^,^^37^ we hypothesised that microglia function may be altered cell-intrinsically in SPG11. Indeed, we found a more pronounced activation of microglia in response to LPS in young asymptomatic *Spg11* KO mice. Activated microglia can release inflammatory cytokines and trigger the recruitment of lymphocytes, further driving neuro-inflammation^60^^,^^61^ thus explaining a previous report that *Spg11* KO mice display substantial neuroinflammation with microgliosis and T-lymphocyte recruitment.^14^

LPS triggers the formation of inflammasomes, which control the activation of inflammatory caspases, in different cell types including microglia. While canonical inflammasomes are composed of Caspase-1, ASC, and a sensor protein such as NLRP3,^62^ non-canonical inflammasomes use Caspase-4 or -5 in humans and Caspase-11 in mice. While the inflammatory response did not differ upon activation of the canonical pathway, the response was strongly enhanced upon activation of the non-canonical inflammasome in *Spg11* KO mice. A selective increase of the response of the non-canonical inflammasome was also evident for BMDMs. The priming signal of the inflammatory response was not affected, because the release of TNF, a direct target of NF-κB, did not differ between genotypes. Importantly, these findings also applied to MDMs from patients with loss-of-function mutations of *SPG11*.

The non-canonical inflammasome plays a major role in the clearance of invading pathogens and signalling to neighbouring cells by mediating the release of alarmins, DAMPs and canonical NLRP3 inflammasome-dependent cytokines.^63^ Consistently, the inflammatory response to a low dose of LPS (5 mg/kg body weight) was strongly increased and the mortality in response to a high dose (20 mg/kg body weight) drastically higher in *Spg11* KO mice. In agreement, boosting the non-canonical inflammasome by transgenic expression of Caspase-4 rendered mice more susceptible to LPS-induced shock, even in the absence of Caspase-1 and Caspase-11.^64^^,^^65^ In contrast, disruption of TLR-4–thereby preventing the priming and activation of the inflammasome–rendered mice more resistant to endotoxic shock upon injection of a high dose of LPS.^48^^,^^66^ Likewise, disruption of either Caspase-1, Caspase-11 or other components of the inflammasome machinery such as ASC and NLRP3 increased the resistance towards LPS-induced shock.^48^^,^^49^^,^^67^^,^^68^

In light of our *in vivo* results and because the inflammatory response of MDMs from patients with SPG11 was strongly increased in response to the activation of the non-canonical inflammasome, it is tempting to speculate that patients with SPG11 might be more susceptible for organ dysfunction upon infection with Gram-negative bacteria, in particular sepsis-associated encephalopathy, a frequent complication of sepsis presenting with delirium, coma, and increased mortality.^69^ However, a systematic evaluation of inflammatory episodes in the history of patients with SPG11 is so far missing and challenging because of the variability of the disorder, with some patients having a slowly progressive and even static course and some progressing more rapidly.^70^ Survivors of sepsis often show neurocognitive impairment,^71^ similarly severe infections may put patients with SPG11 at risk for an accelerated neurodegeneration. This may also apply for patients suffering from SPG48 and even SPG15, because the respective proteins act in a complex with SPG11. Indeed, a pronounced expansion of disease associated microglia and CD8+ effector T cells prior to neuron loss was reported for *Spg15* KO mice.^72^

The hyperactivity of the non-canonical inflammasome is a likely driver of neuro-inflammation and disease progression in SPG11 and SPG48 and possibly SPG15. This is supported by previous reports that the expression of Caspase-11 preceded neuronal defects in mouse models for amyotrophic lateral sclerosis,^73^, ^74^, ^75^ Parkinson's disease^73^ or multiple sclerosis,^74^ while the KO of Caspase-11 attenuated autoimmune encephalomyelitis (EAE) in mice.^74^ Due to the hyper-inflammatory state, patients with SPG11 may benefit from reducing the overall inflammatory stress by lifestyle measures and nutritional intervention. Such lifestyle interventions are currently being studied in different neurodegenerative disorders such as e.g. Parkinson's disease.^76^^,^^77^

We found that two subunits of the AP5 complex were strongly diminished in activated BMDMs from *Spg11* KO mice. The AP5 complex is involved in protein sorting and cargo trafficking at late-endosomal and lysosomal compartments.^7^, ^8^, ^9^ In agreement, disruption of either *Spg11* or *Ap5z1* have been shown to result in a defect in autophagy.^10^^,^^11^ Possibly, defective autophagy may compromise the removal of PAMPs, DAMPs or components of the inflammasome and cytokines thus sensitising the inflammasome.^78^ Indeed, loss of ATG16L1, a protein critical for the initiation of autophagy, enhanced the activation of Caspase-1 and increased production of IL-1β and IL-18 in macrophages after endotoxin treatment.^79^ Likewise, depletion of *Atg 7* promoted the production of IL-1β in macrophages.^80^ Why the defect in autophagy should specifically modulate the non-canonical but not the canonical inflammasome, is still elusive. However, inhibition of autophagy with 3-methyl-adenine or disruption of *Atg 5* also specifically promoted only the activation of the non-canonical inflammasome in macrophages.^81^

A limitation of this study is that the exact molecular events leading to the hyper-responsiveness of the non-canonical inflammasome remain elusive, and clinical data are currently insufficient to assess whether patients suffering from SPG11 or SPG48 are prone to infection-related complications.

Taken together, we here show that Spatacsin and Ap5z1 loss-of-function leads to the hyperactivity of the non-canonical inflammasome. Loss-of-function of either one thus promotes neuro-inflammation, which may perpetuate disease progression. Our findings provide mechanistic insights why immunomodulation significantly attenuated disease progression in a mouse model of SPG11.^82^ Whether anti-inflammatory intervention may prove useful in patients is still unclear.

## Contributors

M.A.A.: Conceptualisation, methodology, investigation and writing of original draft, reviewing, editing, M.G., A.H.: conceptualisation, methodology and analysis, M.A.A: animal experiments, immunofluorescence staining, immunoblots, ELISAs and LDH assays, M.G.: immunofluorescence staining and analysis, A.H.: animal experiments, immunoblots and ELISAs, A.S., L.T.: *in vitro* infections, P.B., A.T.P.: animal experiments, editing, R.H., D.W.: mass spectrometry, A-K. R., R.S.: providing patient samples, J.F.: supervision, editing, M.B.: Supervision, writing, editing, C.A.H: initiation, conceptualisation, writing of original draft, reviewing, editing, supervision.

All authors have read and approved the final version of the manuscript. M.A.A. and C.A.H. have accessed and verified the underlying data.

## Data sharing statement

Materials and associated protocols are available upon request from the corresponding authors.

The mass spectrometry proteomics data have been deposited to the ProteomeXchange Consortium via the PRIDE^33^ partner repository with the dataset identifiers: PXD065699.

## Declaration of interests

The authors declare no conflict of interests.

## Appendix A Supplementary data

Supplementary Tables

Full blots
