# Supplementary material for: Hyperactivity of the non-canonical inflammasome in SPG11 and SPG48
Source: eBioMedicine. 2025 Oct 24;121:105985. doi: 10.1016/j.ebiom.2025.105985 (PMC12595280; doi:10.1016/j.ebiom.2025.105985)
Supplement: Supplementary Figures [file mmc1.pdf]

Supplemental Figures

Hyperactivity of the non-canonical inflammasome in SPG11 and SPG48

Muhammad Awais Afzal[1,9], Mohamed Ghait[2,10], Adeela Hussain[1], Anke Siegmund[3], Lorena Tuchscherr[3], Petra Babic[4], Adrian T. Press[4,11], Robert Hardt[5,12], Dominic Winter[6,12], Annekathrin Rödiger[7,13], Rebecca Schüle[8], Jens Fielitz[2,10], Michael Bauer[4], Christian Andreas Hübner[1,13]

[1] Institute of Human Genetics, Jena University Hospital, Friedrich Schiller University, Am Klinikum1, 07747 Jena, Germany

[2] DZHK (German Center for Cardiovascular Research), partner site Greifswald, 17475 Greifswald, Germany

[3] Institute of Medical Microbiology, Jena University Hospital, 07747 Jena, Germany

[4] Department of Anesthesiology and Intensive Medicine, Jena University Hospital, 07747 Jena, Germany

[5] Seer Bio GmbH - STAC Europe, Venusberg-Campus 1, 53127 Bonn, Germany

[6] Department Metabolism, Senescence and Autophagy, Research Center One Health Ruhr, University Alliance Ruhr, University Hospital Essen, Medical Faculty, University of Duisburg-Essen, 45147 Essen, Germany

[7] Department of Neurology, Neuromuscular Center, Jena University Hospital, Germany

[8] Division of Neurodegenerative Diseases and Movement Disorders, Department of Neurology, Heidelberg University Hospital and Faculty of Medicine, Heidelberg, Germany

[9] Center for Sepsis Control and Care (CSCC), Jena University Hospital, 07747 Jena, Germany

[10] Department of Internal Medicine B, Cardiology, University Medicine Greifswald, 17475 Greifswald, Germany

[11] Friedrich Schiller University, Medical Faculty, Kastanienstr. 1, 07747 Jena

[12] Institute of Biochemistry and Molecular Biology, University of Bonn, 53115 Bonn, Germany

[13] Center for Rare Diseases, University Hospital Jena, Friedrich Schiller University, Am Klinikum1, 07747 Jena, Germany

Correspondence:

Christian Andreas Hübner

Institut für Humangenetik, Universitätsklinikum Jena, Friedrich Schiller Universität,

Am Klinikum 1

07747 Jena

Christian.huebner@med.uni-jena.de

Tel. 0049-3641-9396800

Supplementary Figure S1: The priming and the canonical activation of the inflammasome are independent of Spatacsin in bone marrow derived macrophages

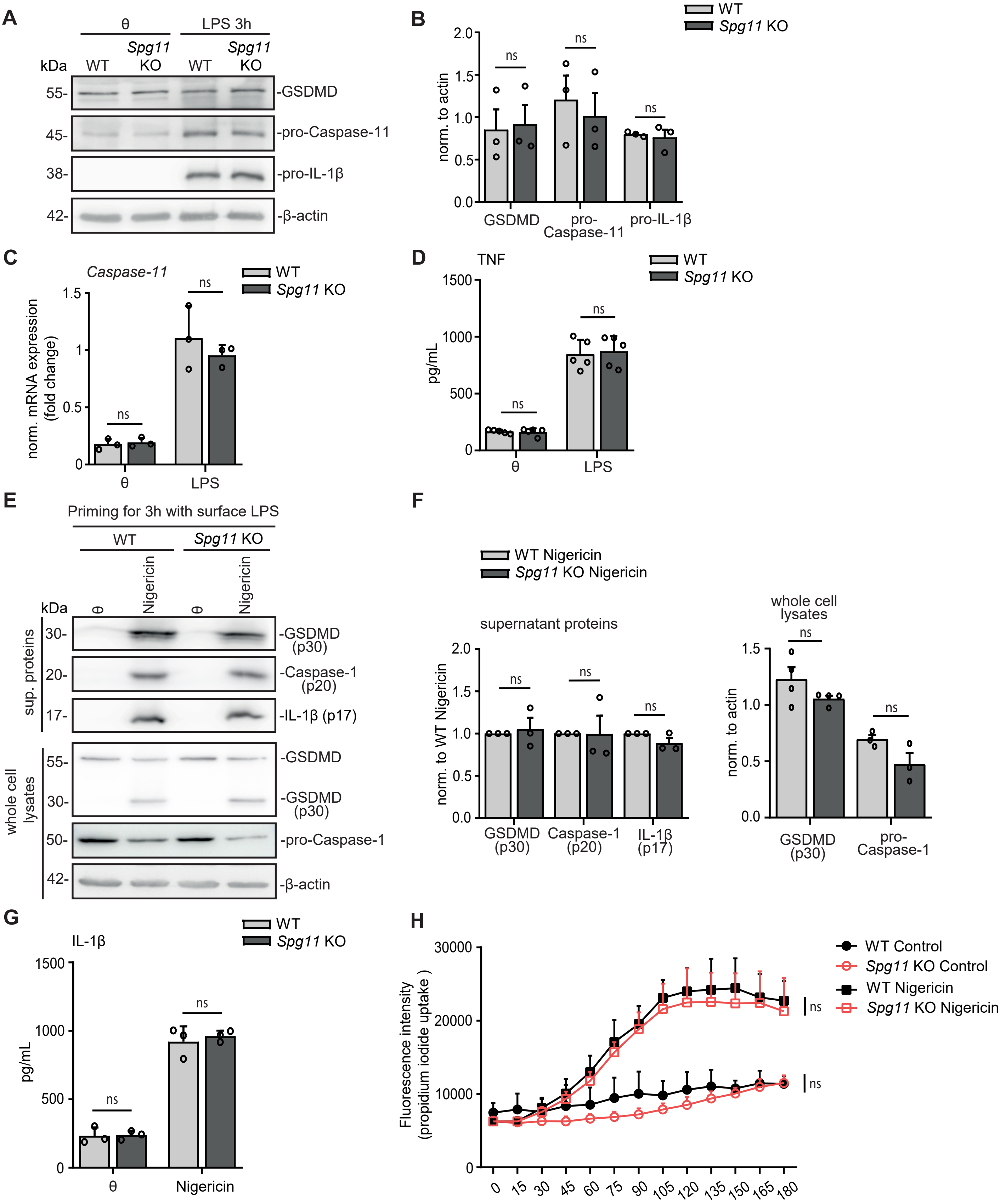

A, B, Immunoblot analyses of BMDM protein lysates. Abundances of GSDMD, pro-Caspase-11, and pro-IL-1β after LPS priming of WT and *Spg11* KO macrophages were measured (n=3 independent experiments; two-sided paired Student's t-test: ns- not significant). **C,** *Caspase-11* transcript abundance assessed by qPCR for both genotypes after LPS priming (n=3 independent experiments; two-way ANOVA followed by Sidak's multiple comparison test: ns- not significant). **D,** TNF release measured by ELISA between genotypes after LPS priming of BMDMs (n=5 independent experiments; two-way ANOVA followed by Sidak's multiple comparison test: ns- not significant). **E, F,** Immunoblot analyses for GSDMD (p30), Caspase-1 (p20), and IL-1β abundances in supernatants from WT and *Spg11* KO LPS-primed BMDMs after nigericin treatment (n=3 independent experiments; two-sided paired Student's t-test: ns- not significant). The GSDMD (p30) and pro-Caspase-1 levels were determined after nigericin treatment in WT and *Spg11* KO LPS-primed BMDMs total protein lysates (n=3-4 independent experiments; two-sided paired Student's t-test: ns- not significant). **G,** The abundance of IL-1β measured by ELISA after nigericin treatment for both genotypes in supernatants from LPS-primed BMDMs (n=3 independent experiments; two-sided paired Student's t-test: ns- not significant). **H,** Cell viability assessed by propidium iodide (PI) uptake for WT and *Spg11* KO LPS-primed BMDMs at different time points after nigericin treatment (n=3 independent experiments; two-way ANOVA followed by Bonferroni post-hoc test: ns- not significant). Quantitative data are shown as mean ± SEM.

Supplementary Figure S2: The activation of the inflammasome is increased in response to infection with Gram-negative bacteria

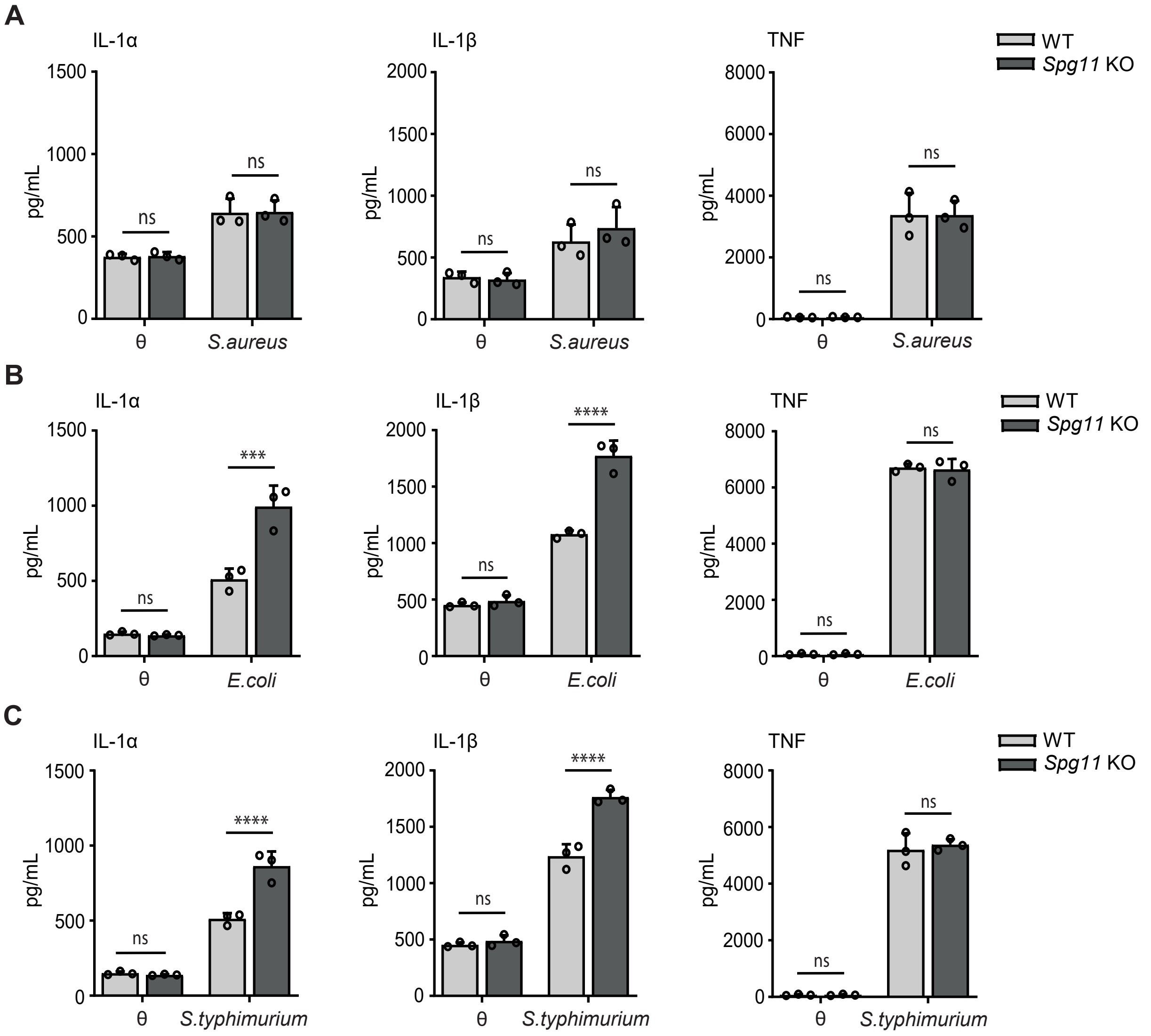

A, Release of IL-1α and IL-1β or TNF upon infection with Gram-positive *S. aureus* (n=3 independent experiments; two-way ANOVA followed by Sidak's multiple comparison test: ns- not significant). **B, C,** IL-1α, IL-1β, and TNF release from WT and S*pg11* KO BMDMs 3 h after infection with either Gram-negative *E. coli* (B) or *S. typhimurium* (C) (n=3 independent experiments; two-way ANOVA followed by Sidak's multiple comparison test: ****p<0.0001, ***p<0.001, ns- not significant). Quantitative data are shown as mean ± SEM.

Supplementary Figure S3: The canonical activation of the inflammasome is independent of Ap5z1

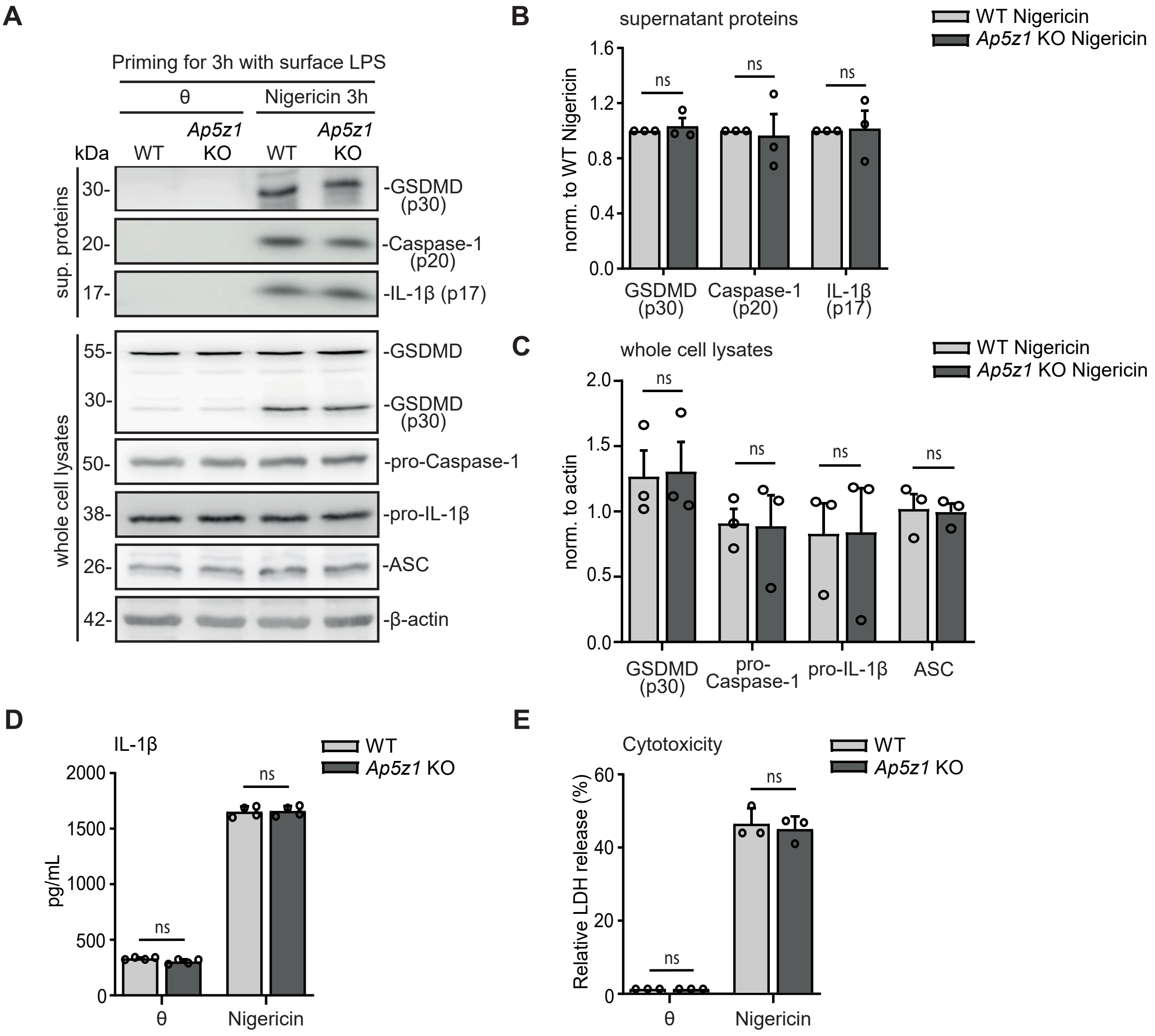

A-C, Cells were primed with extracellular LPS for 3 h and the canonical inflammasome activated by incubation with nigericin for 3 h. Immunoblot analyses of protein lysates and supernatants of BMDMs from WT and *Ap5z1* KO mice (A). GSDMD (p30), Caspase-1 (p20), and IL-1β abundances were quantified in cell supernatants (B). The abundances of GSDMD (p30), pro-Caspase-1, pro-IL-1β, and ASC were measured in whole cell lysates (C) (n=3 independent experiments; two-sided paired Student's t-test: ns- not significant). **D,** The ELISA for IL-1β levels in supernatants of non-activated and activated BMDMs (n=4 independent experiments; two-way ANOVA followed by Sidak's multiple comparison test: ns- not significant). **E,** Relative LDH release (n=3 independent experiments; two-way ANOVA followed by Sidak's multiple comparison test: ns- not significant). Quantitative data are shown as mean ± SEM.
