# Supplementary material for: Hyperactivity of the non-canonical inflammasome in SPG11 and SPG48
Source: eBioMedicine. 2025 Oct 24;121:105985. doi: 10.1016/j.ebiom.2025.105985 (PMC12595280; doi:10.1016/j.ebiom.2025.105985)
Supplement: Supplementary Tables [file mmc2.pdf]

Supplemental Tables

Hyperactivity of the non-canonical inflammasome in SPG11 and SPG48

Muhammad Awais Afzal[1,9], Mohamed Ghait[2,10], Adeela Hussain[1], Anke Siegmund[3], Lorena Tuchscherr[3], Petra Babic[4], Adrian T. Press[4,11], Robert Hardt[5,12], Dominic Winter[6,12], Annekathrin Rödiger[7,13], Rebecca Schüle[8], Jens Fielitz[2,10], Michael Bauer[4], Christian Andreas Hübner[1,13]

[1] Institute of Human Genetics, Jena University Hospital, Friedrich Schiller University, Am Klinikum1, 07747 Jena, Germany

[2] DZHK (German Center for Cardiovascular Research), partner site Greifswald, 17475 Greifswald, Germany

[3] Institute of Medical Microbiology, Jena University Hospital, 07747 Jena, Germany

[4] Department of Anesthesiology and Intensive Medicine, Jena University Hospital, 07747 Jena, Germany

[5] Seer Bio GmbH - STAC Europe, Venusberg-Campus 1, 53127 Bonn, Germany

[6] Department Metabolism, Senescence and Autophagy, Research Center One Health Ruhr, University Alliance Ruhr, University Hospital Essen, Medical Faculty, University of Duisburg-Essen, 45147 Essen, Germany

[7] Department of Neurology, Neuromuscular Center, Jena University Hospital, Germany

[8] Division of Neurodegenerative Diseases and Movement Disorders, Department of Neurology, Heidelberg University Hospital and Faculty of Medicine, Heidelberg, Germany

[9] Center for Sepsis Control and Care (CSCC), Jena University Hospital, 07747 Jena, Germany

[10] Department of Internal Medicine B, Cardiology, University Medicine Greifswald, 17475 Greifswald, Germany

[11] Friedrich Schiller University, Medical Faculty, Kastanienstr. 1, 07747 Jena

[12] Institute of Biochemistry and Molecular Biology, University of Bonn, 53115 Bonn, Germany

[13] Center for Rare Diseases, University Hospital Jena, Friedrich Schiller University, Am Klinikum1, 07747 Jena, Germany

Correspondence:

Christian Andreas Hübner

Institut für Humangenetik, Universitätsklinikum Jena, Friedrich Schiller Universität,

Am Klinikum 1

07747 Jena

Christian.huebner@med.uni-jena.de

Tel. 0049-3641-9396800

Supplementary Table 1: Demographic data from patients of SPG11 and healthy controls

Sample ID	Causative Gene	Causative Mutations	Sex	Age at biopsy (Years)
Control 1	Healthy control	-	female	22
Control 2	Healthy control	-	male	30
Control 3	Healthy control	-	female	24
Control 4	Healthy control	-	male	26
Control 5	Healthy control	-	female	28
Patient 1	SPG11	Allele 1: c.4307_4308del, p.Gln1436Argfs*7)<br><br>Allele 2: c.1348dup, p.lle450Asnfs*26)	female	19
Patient 2	SPG11	Allele 1: c.4307_4308delAA, p.Q1436RfsX7<br><br>Allele 2: c.6890_6892delTGA, p.12298del (p.12297Lfs12298del)	male	30
Patient 3	SPG11	Allele 1: c.[4790G>A, p.Trp1597*;<br><br>Allele 2: c.5093T>A, p.Leu1698*	female	26
Patient 4	SPG11	Allele 1: c.5757_5758del, p.Glu1921Serfs*2;<br><br>Allele 2: c.5989_5992del, p.Leu1997Metfs*60	male	27
Patient 5	SPG11	Allele 1: c.4198C>T; p.Gln1400*;<br><br>Allele 2: c.6642dupC; p.Ser2215Glnfs*2	female	21

Supplementary Table 2: Primary and secondary antibodies

**Primary Antibody**	**Dilution**	**Cat. #, RRID**	**Manufacturer**
ASC	1:1000	NBP1-78977, RRID:AB_11015255	Novus Biologicals
ASC	1:1000	SAB4501315, RRID:AB_10746058	Sigma-Aldrich
β-actin	1:10,000	ab6276, RRID:AB_2223210	Abcam
Caspase-1	1:1000	ab179515, RRID:AB_2884954	Abcam
Caspase-11 (17D9)	1:1000	NB120-10454, RRID:AB_788441	Novus Biologicals
Gasdermin D (EPR19828)	1:1000	ab209845, RRID:AB_2783550	Abcam
GFAP (Glial Fibrillary Acidic Protein)	1:1000	MAB360, RRID:AB_2275415	Millipore
Hoechst 33258	1:10,000	H3569, RRID:AB_2651133	Invitrogen
IBA-1	1:5000	019-19741, RRID:AB_839504	Fujifilm
IL-1β (3A6)	1:1000	12242S, RRID:AB_2715503	Cell Signaling Technology
**Secondary Antibody**	**Dilution**	**Cat. #, RRID**	**Manufacturer**
Goat anti-Rabbit IgG, Alexa Fluor 546	1:5000	A11035, RRID:AB_143051	Thermo Fisher
Goat anti-Rabbit IgG, Alexa Fluor 555	1:1000	A-21428, RRID:AB_141784	Thermo Fisher
Mouse IgG HRP Linked F(ab′)2	1:6000	GENA9310, RRID: AB_772193	Merck
Rabbit IgG HRP Linked F(ab′)2	1:6000	GENA9340, RRID: AB_772191	Merck
Goat anti-Rat IgG HRP	1:5000	31470, RRID:AB_228356	Thermo Fisher
Streptavidin-HRP Conjugate	1:6000	GERPN1231	Merck

Supplementary Table 3: Kits

Name of Kit	Product number, Company
Mouse IL-1α DuoSet ELISA	DY400-05, R&D Systems
Mouse IL-1β DuoSet ELISA	DY401-05, R&D Systems
Mouse IL-18 DuoSet ELISA	DY7625-05, R&D Systems
Mouse TNF-α DuoSet ELISA	DY410-05, R&D Systems
Human IL-1β ELISA kit	CHC1213, Invitrogen
Human IL-18 ELISA kit	BMS267-2MST, Invitrogen
LDH-Cytox™ Assay Kit	426401, BioLegend
Pierce™ BCA Protein Assay Kit	23225, Thermo Fisher
SuperScript™ III reverse transcription kit	18080-044, Invitrogen

Supplementary Table 4: qPCR Primers

Primer name	Sequence (5'-3')
*Caspase-11*_F	AGA GGT GGG AAC TCT GGA GAA
*Caspase-11*_R	AGC CTC CTG TTT TGT CTC GG
*β-actin*_F	CTA AGG CCA ACC GTG AAA AG
*β-actin*_R	ACC AGA GGC ATA CAG GGA CA

Supplementary Table 5: Clinical Severity Score (CSS)

Clinical Severity Score (CSS)						
CSS	Quality	Evaluation Unit	Criteria			
			Activity	Reaction to external stimulus	Posture	Symptoms
1	No signs of illness (1), <5 points	1 point/criteria	Active, strong	Curious, fast and immediate reaction	normal	none
2	Low-grade illness (2) <9 points	2 points/criteria	Lowered activity, partial interrupted movements	Reduced attentiveness, adequate reaction	Slightly hunched, piloerection	diarrhoea
3	Mid-grade illness (3) <13 points	3 points/criteria	Slow, drowsy, problem in movement	Reduced and delayed reaction	Hunched	Bodyweight reduced by 10%/24 h<br><br>Body temperature altered by 1-2ºC
4	High-grade illness (4) ≥ 13 points	4 points/criteria	Lethargic, no movement	None	Strongly hunched	Bodyweight reduced by >10%/24 h<br><br>Body temperature altered by >2ºC
