# Supplementary material for: Hyperactivity of the non-canonical inflammasome in SPG11 and SPG48
Source: eBioMedicine. 2025 Oct 24;121:105985. doi: 10.1016/j.ebiom.2025.105985 (PMC12595280; doi:10.1016/j.ebiom.2025.105985)

Uncropped full Western Blots

Hyperactivity of the non-canonical inflammasome in SPG11 and SPG48

Afzal et al., 2025

Figure 4 E
Whole cell lysates, *Spg11* KO macrophages
GSDMD

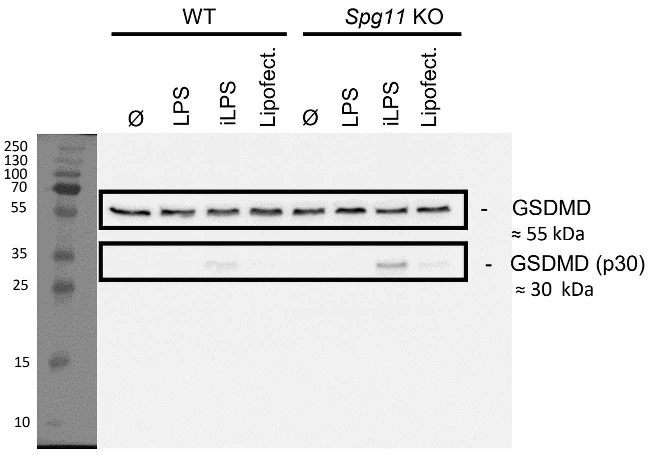

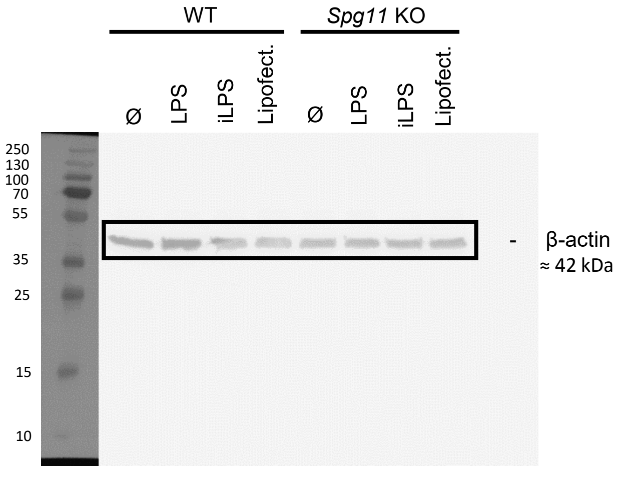

Figure 4 E
Whole cell lysates, *Spg11* KO macrophages
pro-Caspase-1

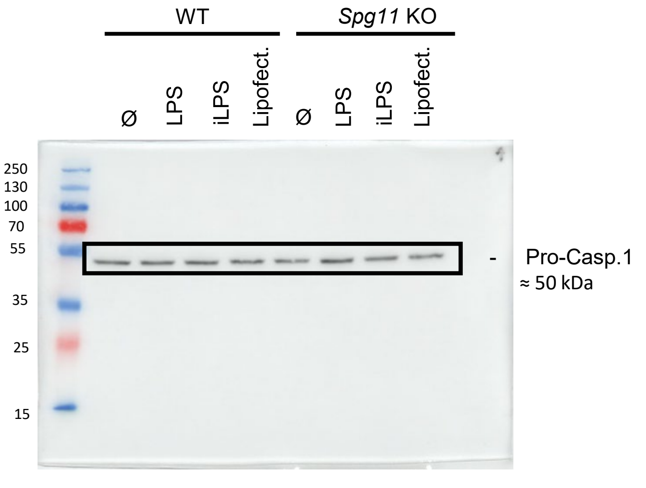

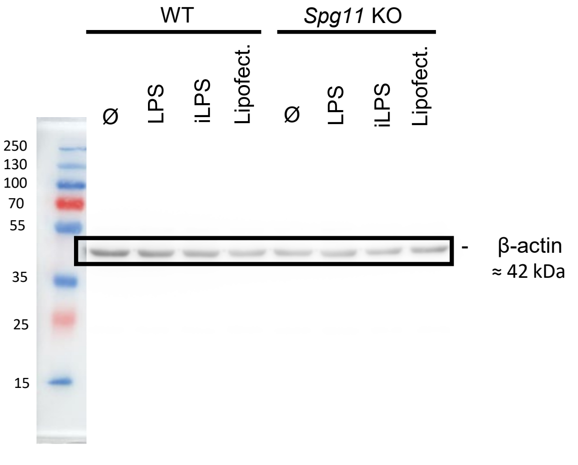

Figure 4 E
Sup. proteins, *Spg11* KO macrophages
GSDMD (p30)

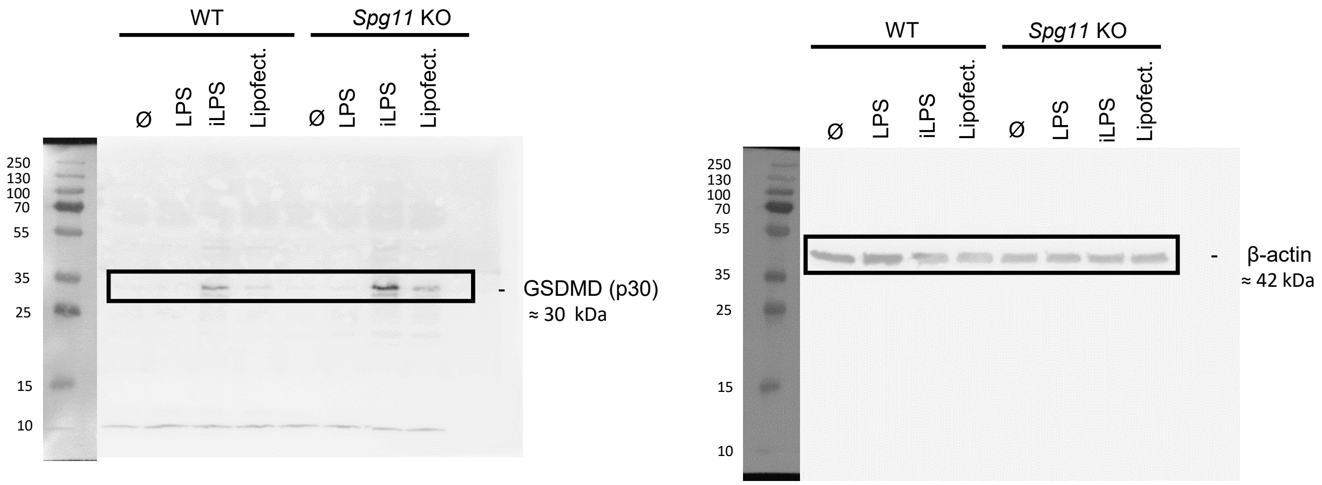

Figure 4 E
Sup. proteins, *Spg11* KO macrophages
Caspase-1 (p20)

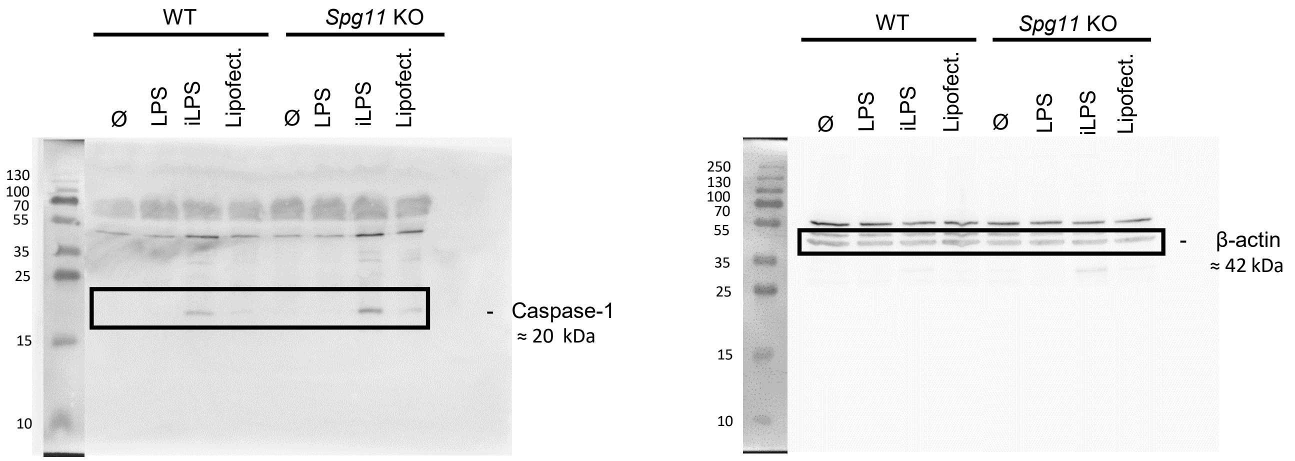

Figure 4 E
Sup. proteins, *Spg11* KO macrophages
IL-1β (p17)

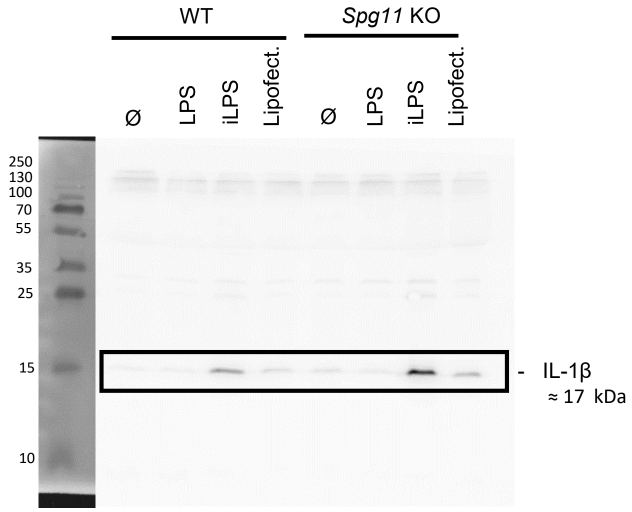

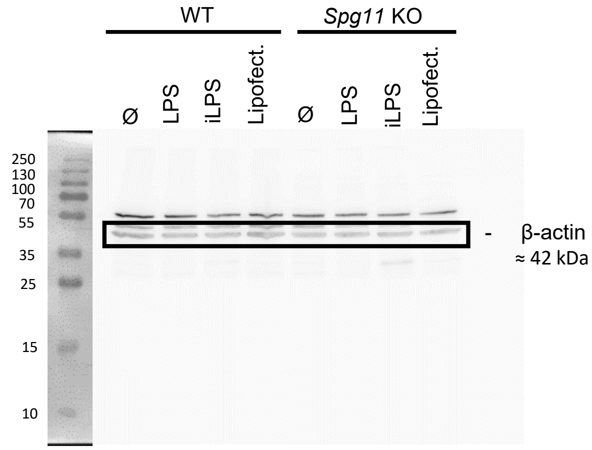

Figure 6 E
Whole cell lysates, *Ap5z1* KO macrophages
GSDMD

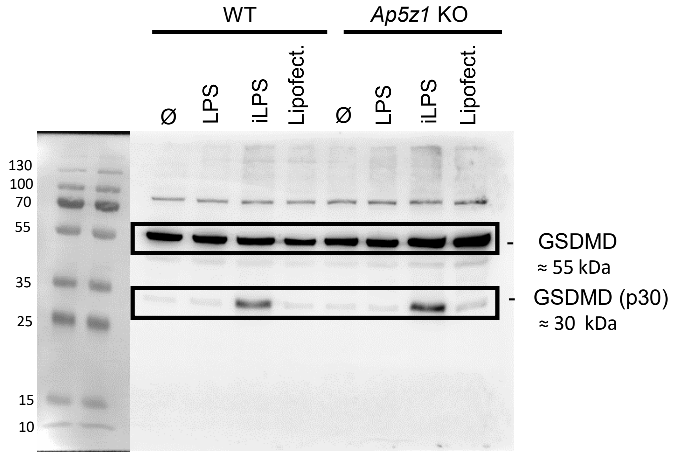

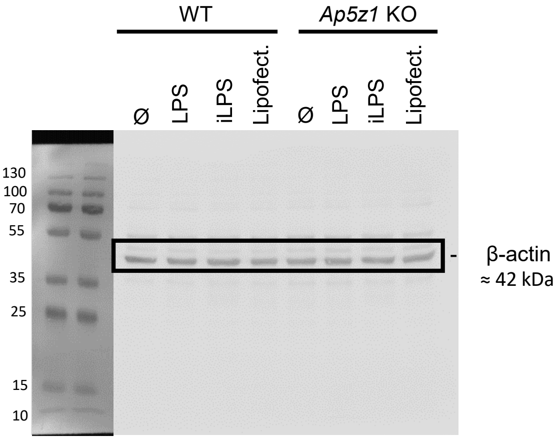

Figure 6 E
Whole cell lysates, *Ap5z1* KO macrophages
pro-Caspase-1

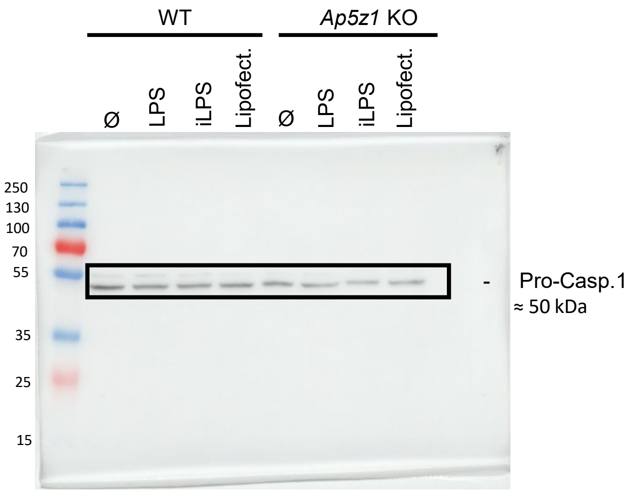

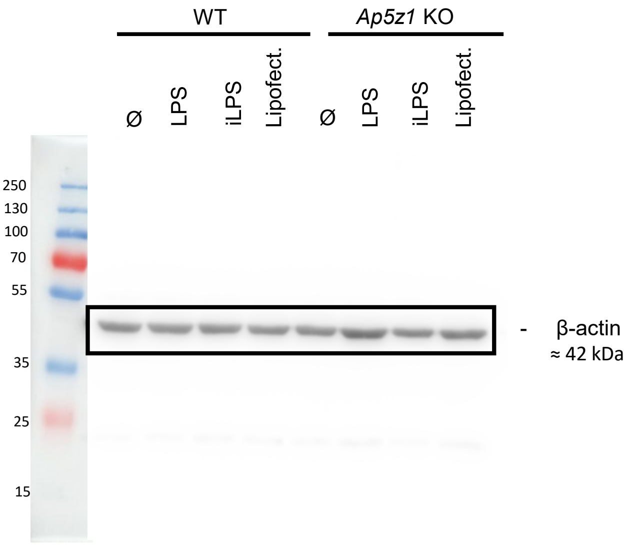

Figure 6 E
Sup. proteins, *Ap5z1* KO macrophages
GSDMD (p30)

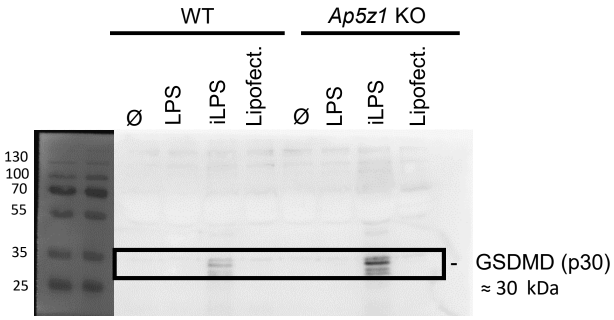

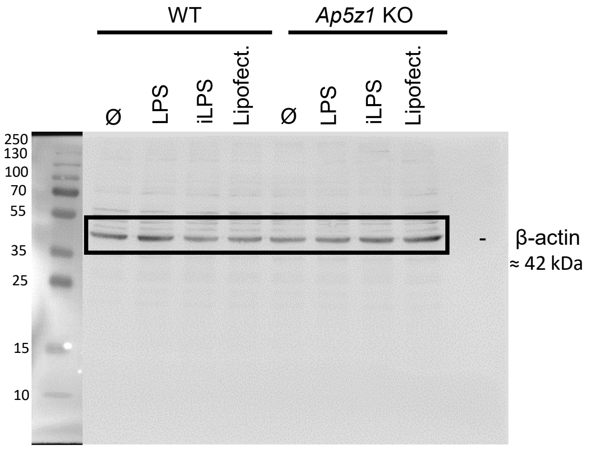

Figure 6 E
Sup. proteins, *Ap5z1* KO macrophages
Caspase-1 (p20)

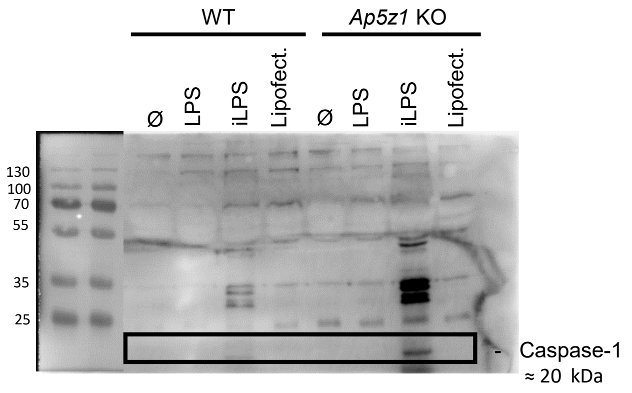

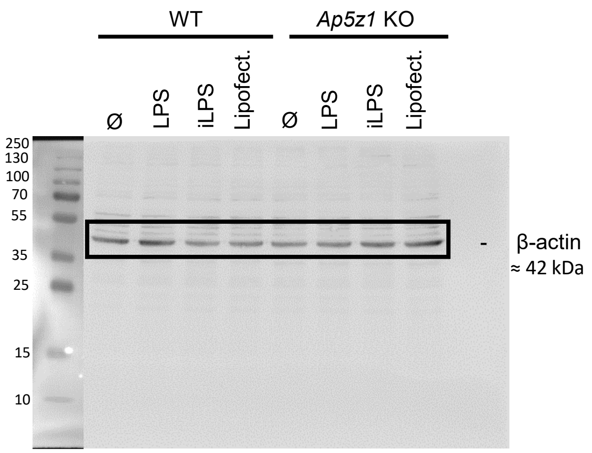

Figure 6 E
Sup. proteins, *Ap5z1* KO macrophages
1L-1β (p17)

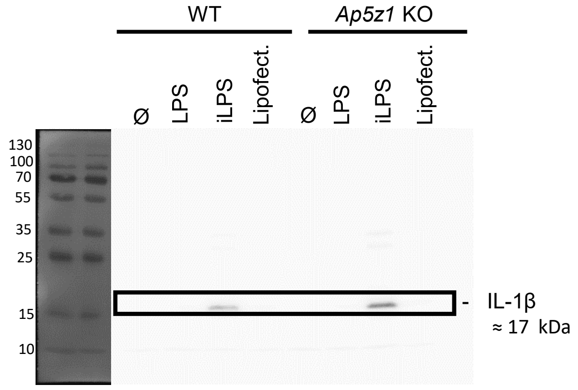

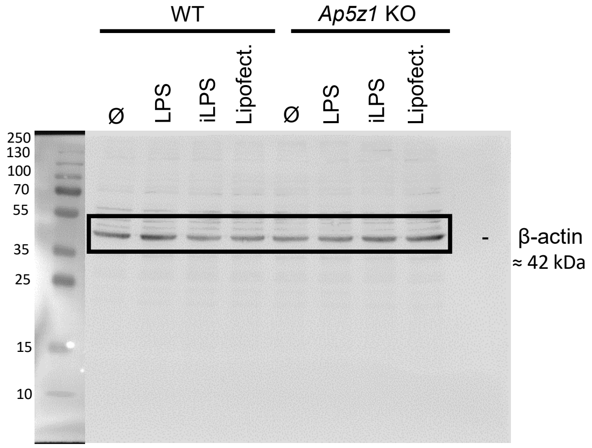

Figure 6 G
Whole cell lysates, *Ap5z1* KO macrophages
GSDMD

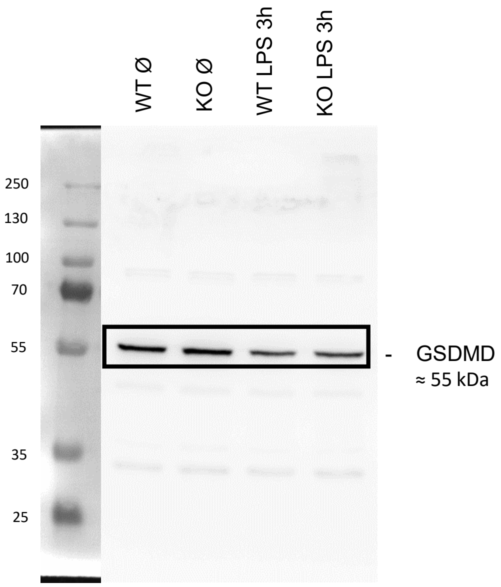

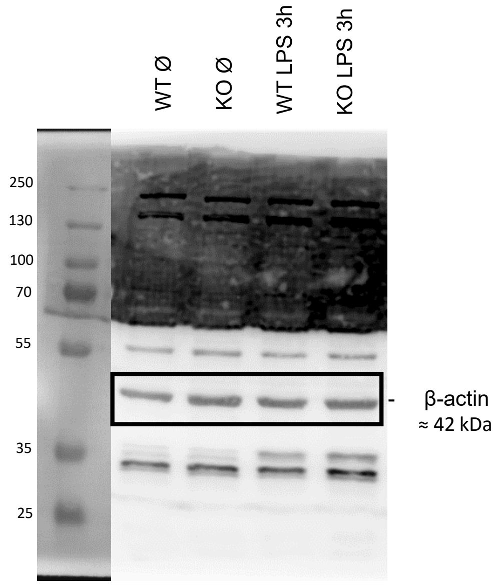

Figure 6 G
Whole cell lysates, *Ap5z1* KO macrophages
pro-Caspase-1

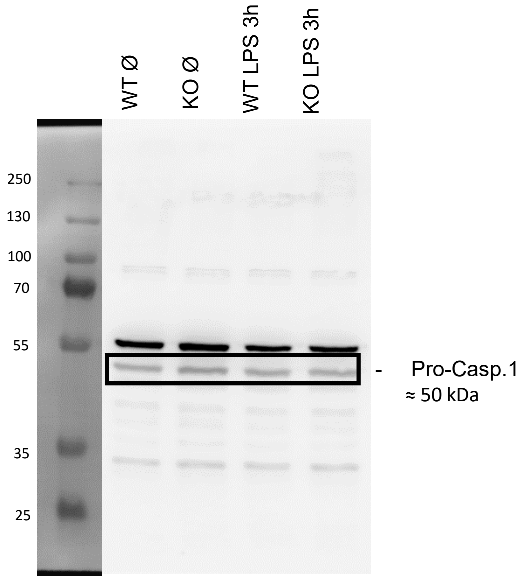

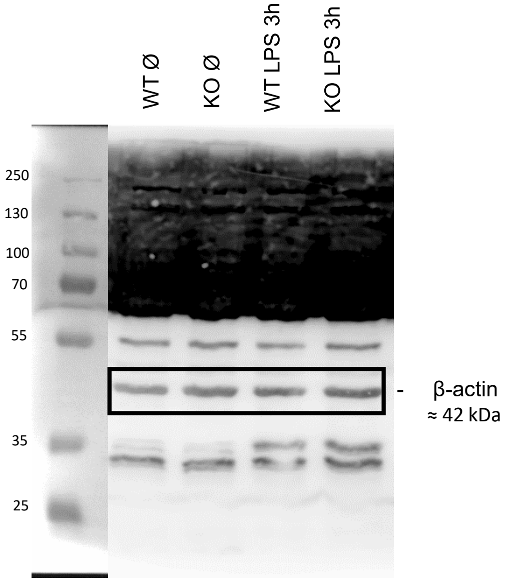

Figure 6 G
Whole cell lysates, *Ap5z1* KO macrophages
pro-IL-1β

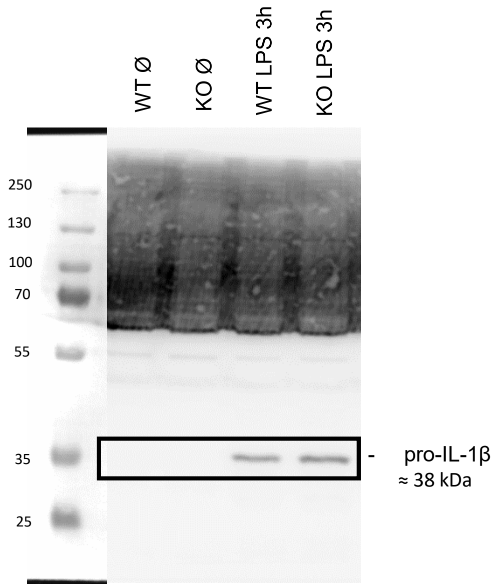

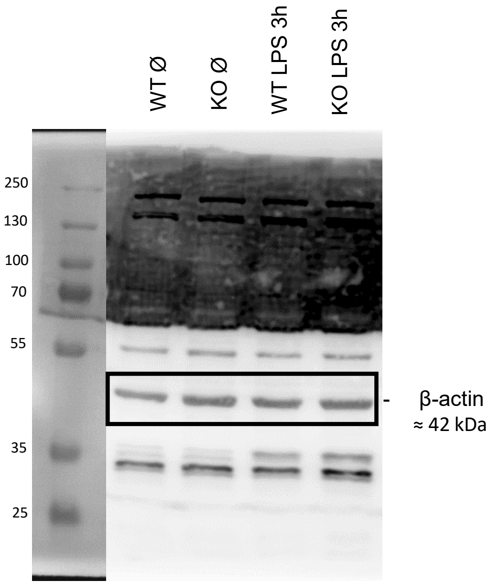

Figure 7 I
Spleen tissue lysates – LPS-induced mice
GSDMD (p30)

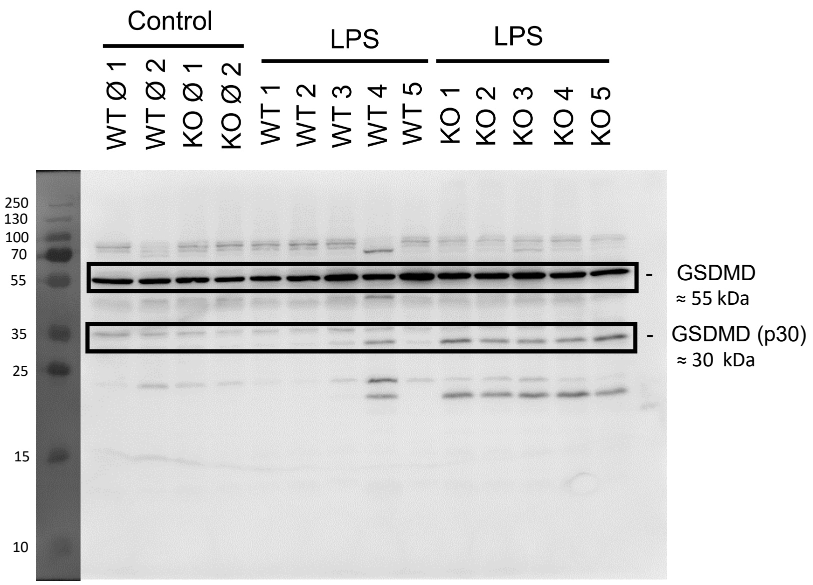

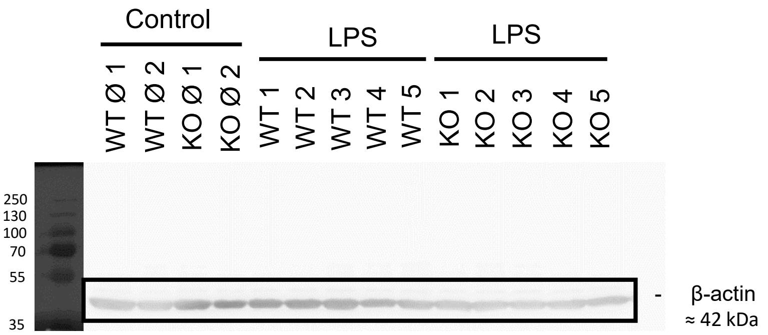

Suppl. Figure S1 A
Whole cell lysates, *Spg11* KO macrophages
GSDMD

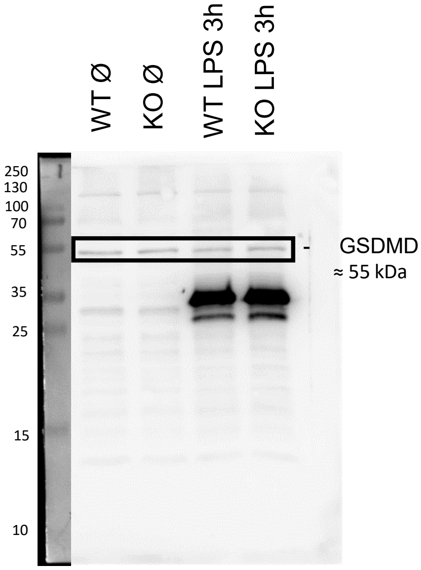

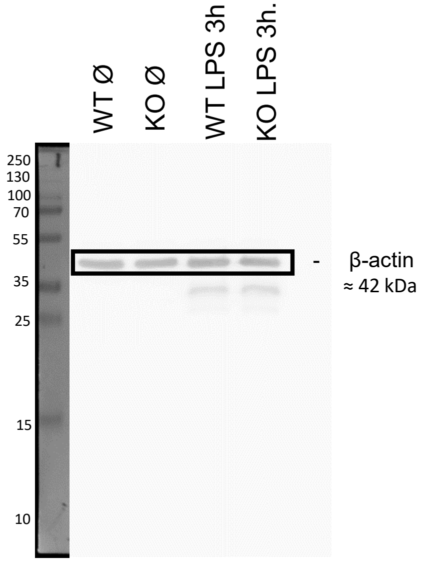

Suppl. Figure S1 A
Whole cell lysates, *Spg11* KO macrophages
pro-Caspase-11

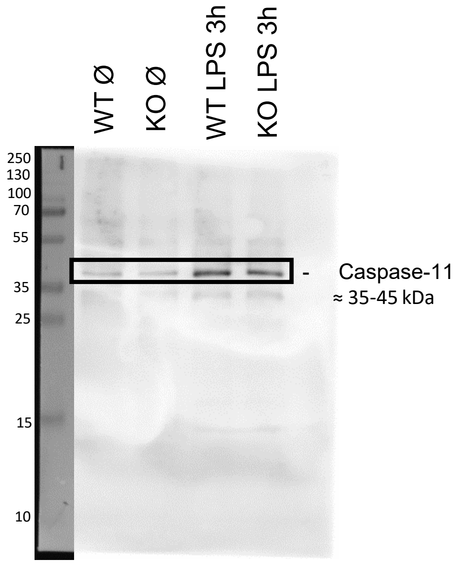

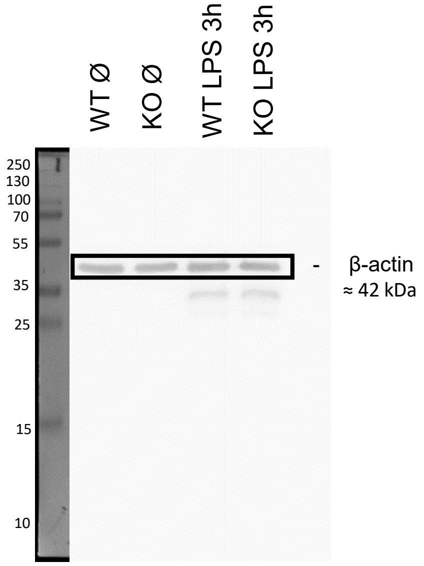

Suppl. Figure S1 A
Whole cell lysates, *Spg11* KO macrophages
pro-IL-1β

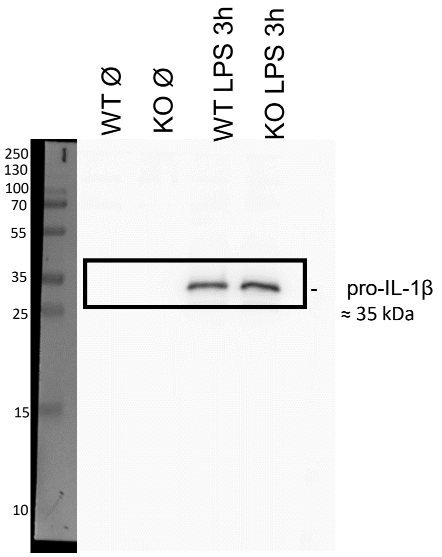

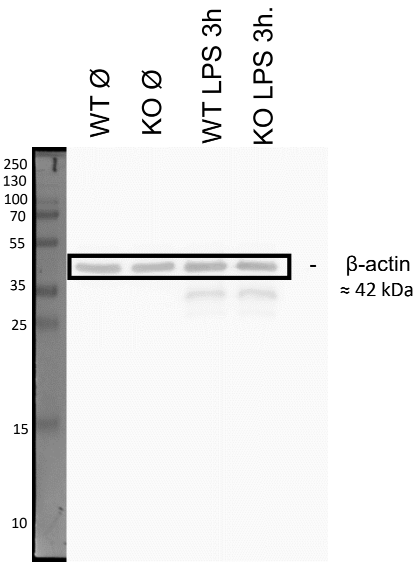

Suppl. Figure S1 E
Whole cell lysates, *Spg11* KO macrophages
GSDMD (p30)

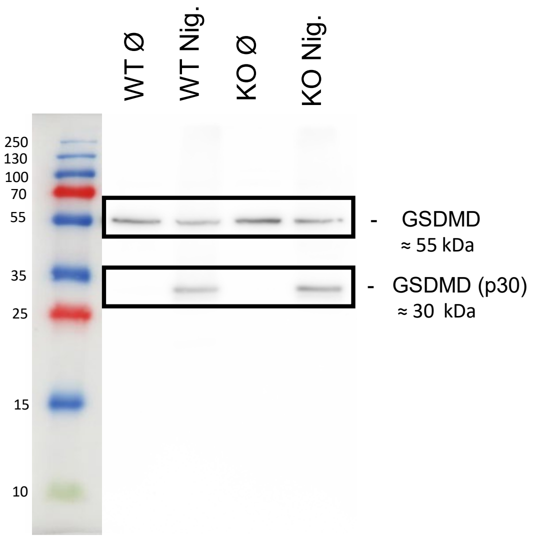

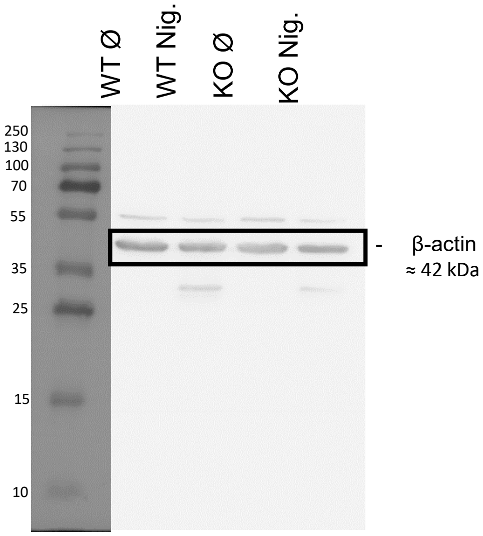

Suppl. Figure S1 E
Whole cell lysates, *Spg11* KO macrophages
pro-Caspase-1

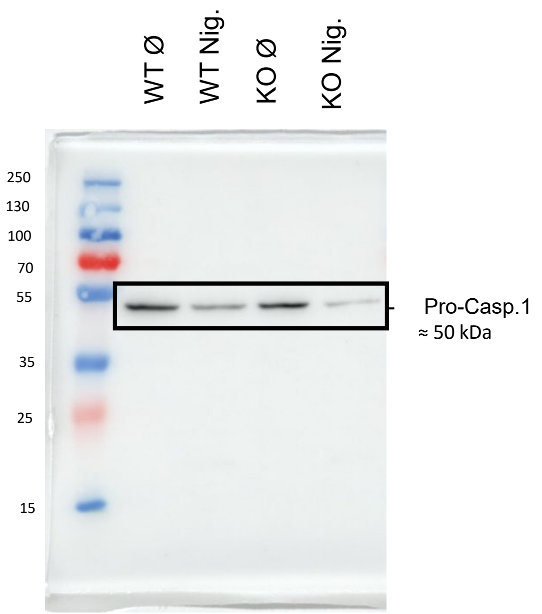

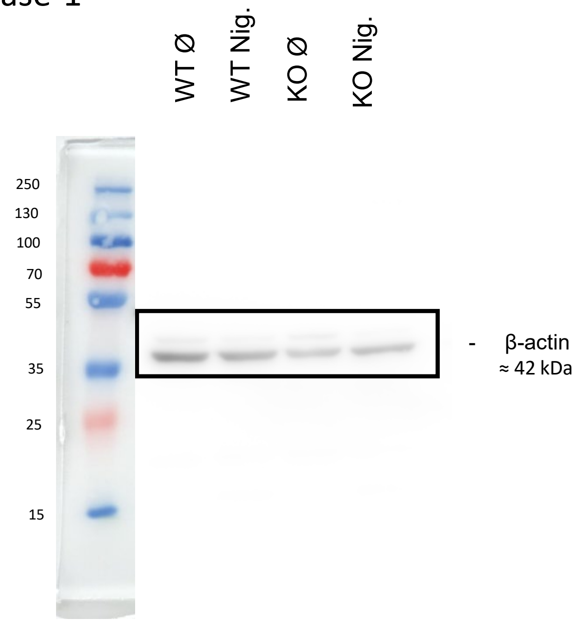

Suppl. Figure S1 E
Supernatant proteins, *Spg11* KO macrophages
GSDMD (p-30)

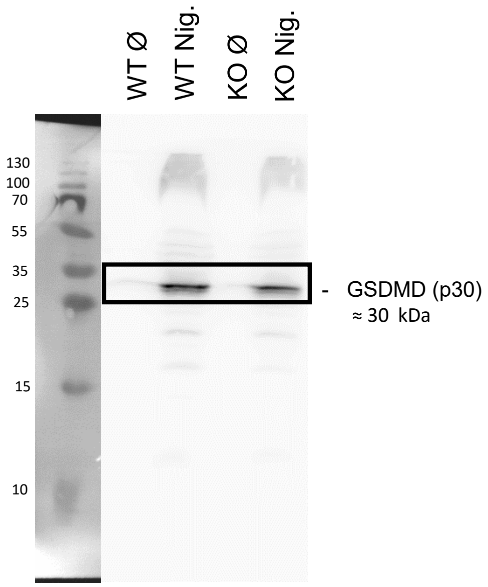

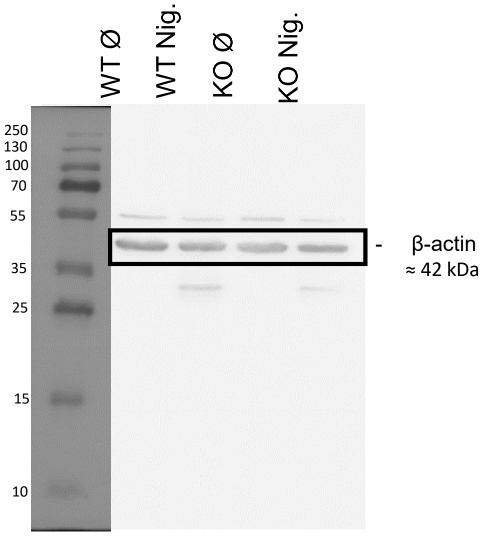

Suppl. Figure S1 E
Supernatant proteins, *Spg11* KO macrophages
Caspase-1 (p20)

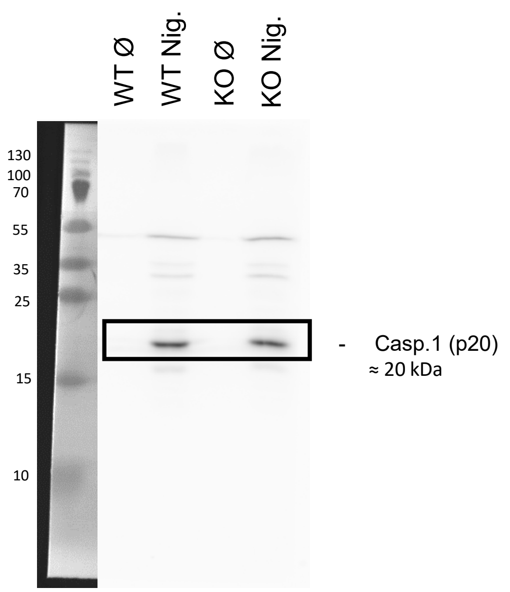

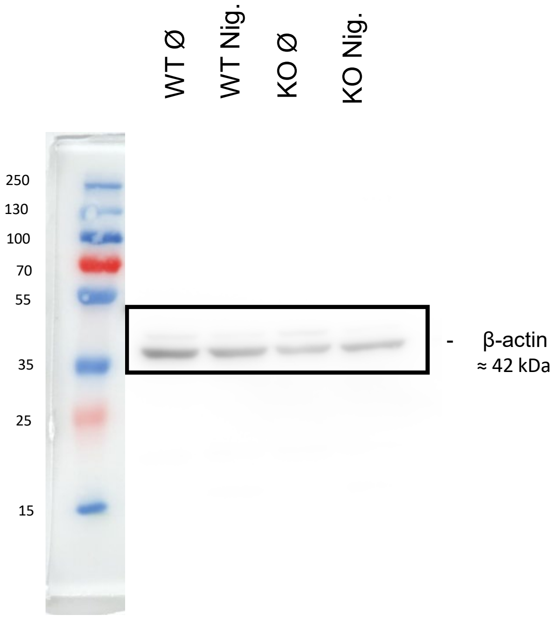

Suppl. Figure S1 E
Supernatant proteins, *Spg11* KO macrophages
IL-1β (p17)

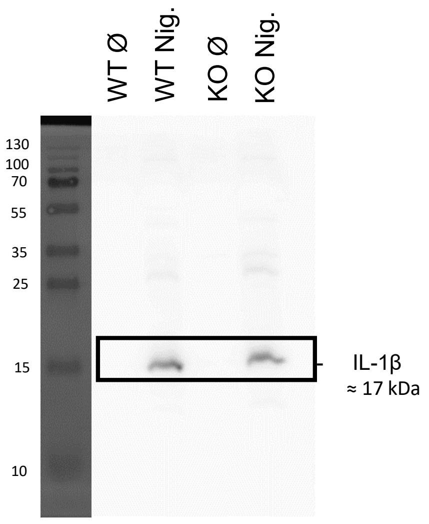

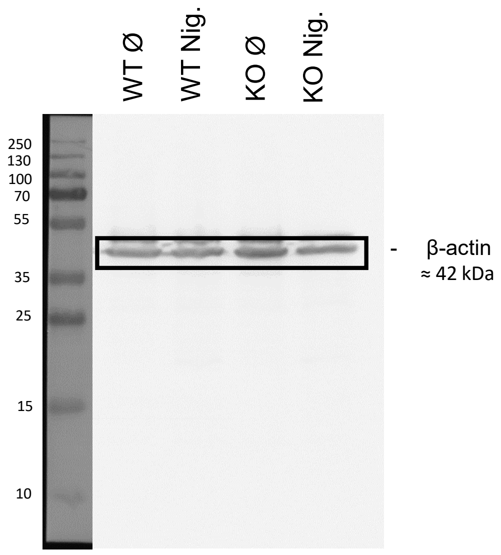

Suppl. Figure S3 A
Whole cell lysates, *Ap5-z1* KO macrophages
GSDMD-p30

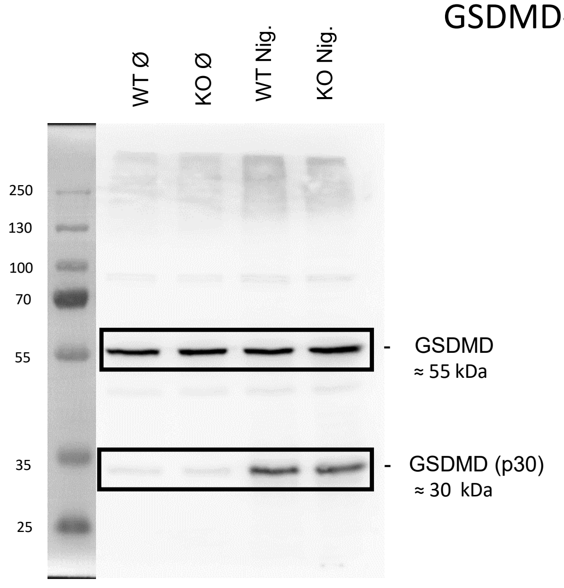

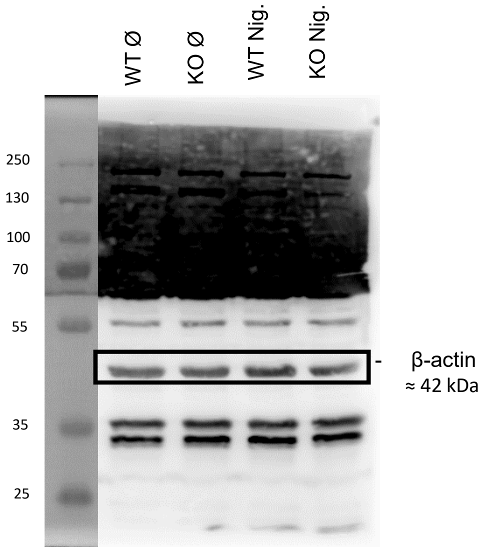

Suppl. Figure S3 A
Whole cell lysates, *Ap5-z1* KO macrophages
Pro-Caspase-1

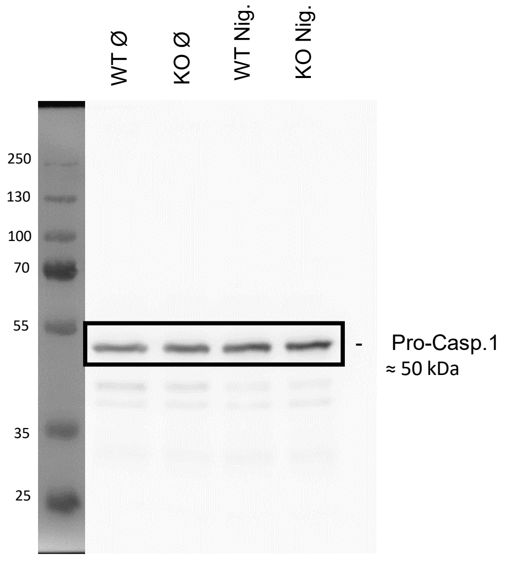

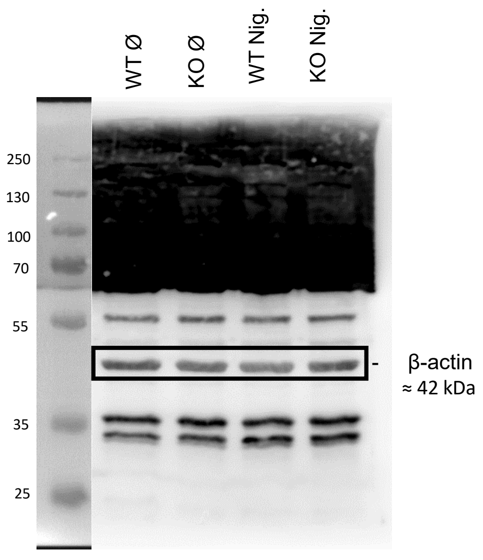

Suppl. Figure S3 A
Whole cell lysates, *Ap5-z1* KO macrophages
Pro-IL-1β

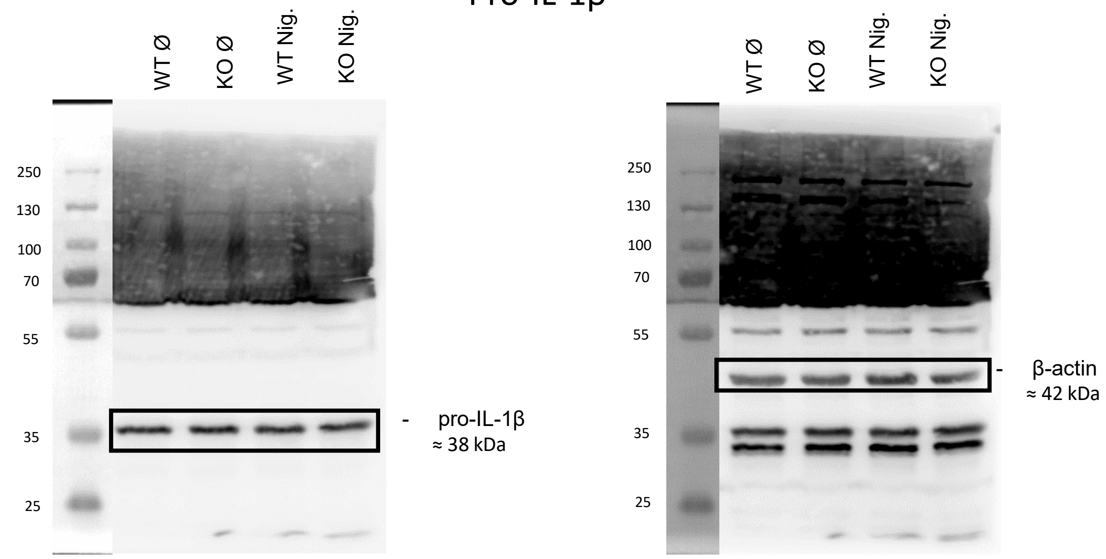

Suppl. Figure S3 A
Whole cell lysates, *Ap5-z1* KO macrophages
ASC

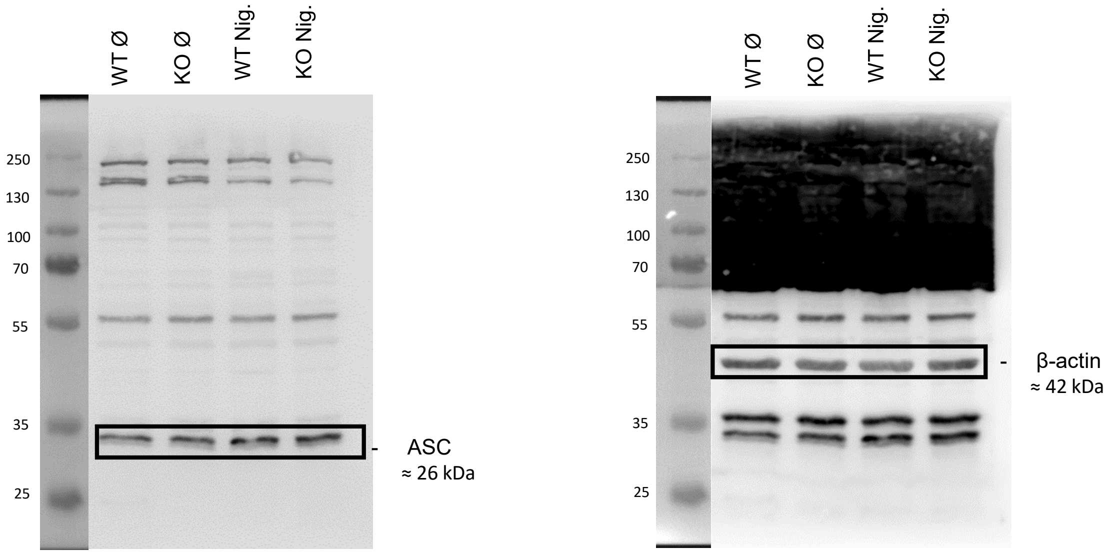

Suppl. Figure S3 A

Supernatant proteins, *Ap5-z1* KO macrophages

GSDMD-p30

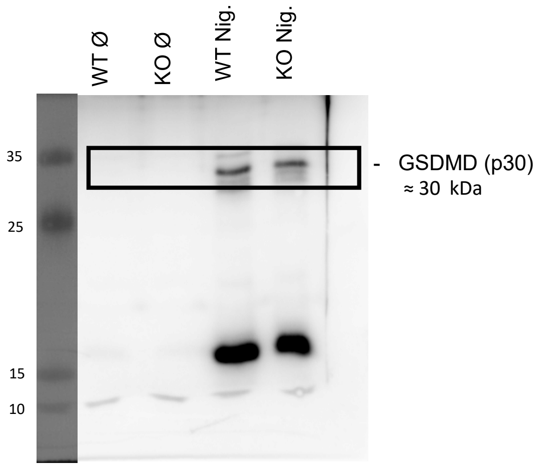

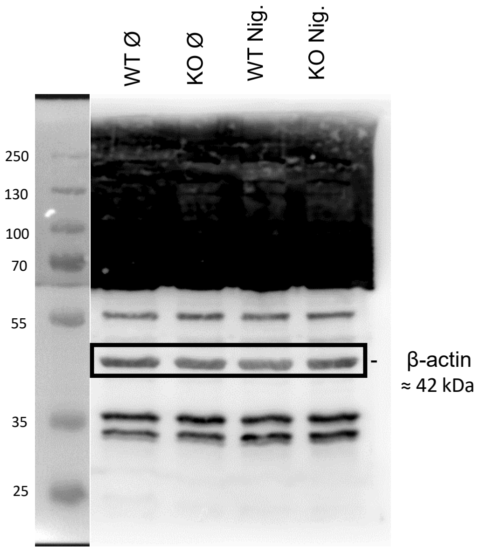

Suppl. Figure S3 A

Supernatant proteins, *Ap5-z1* KO macrophages

Caspase1-p20

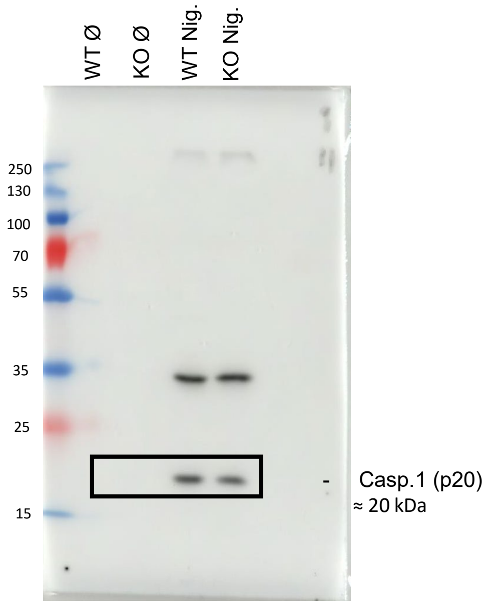

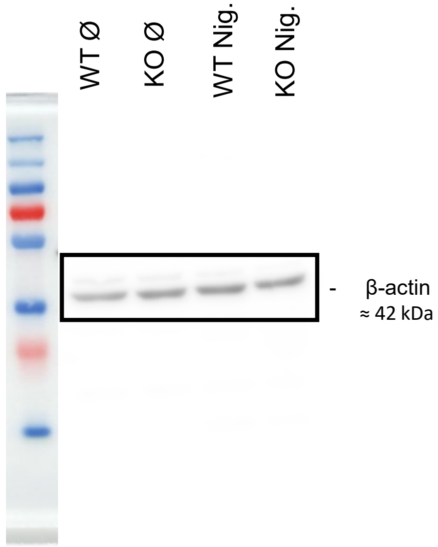

Suppl. Figure S3 A
Supernatant proteins, *Ap5-z1* KO macrophages
IL-1β (p17)

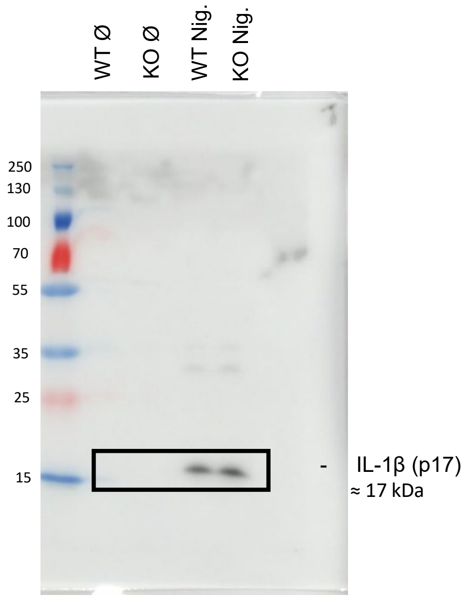

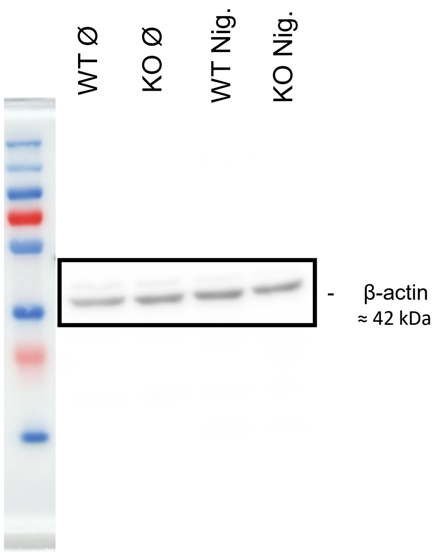

Supplement: Full blots [file mmc3.pdf]
